# The prefrontal operculum, a human-specific hub for the cognitive control of speech

**DOI:** 10.1038/s42003-025-09110-8

**Published:** 2025-12-01

**Authors:** Charles Verstraete, Elsa Lazzaro, Delphine Autran-Clavagnier, Manon Dirheimer, Fadila Hadj-Bouziane, Franck Lamberton, Kep Kee Loh, Emmanuel Procyk, Charles R. E. Wilson, Camille Giacometti, Céline Amiez

**Affiliations:** 1 Univ Lyon, Université Lyon 1, Inserm, Stem Cell and Brain Research Institute, Bron, France; 2 Institut de neuromodulation, Groupe Hospitalo-Universitaire Paris, Psychiatrie et Neurosciences, Université Paris Cité, Paris, France; 3 Inovarion, Paris, France; 4 Integrative Multisensory Perception Action and Cognition Team (ImpAct), INSERM U1028, CNRS UMR5292, Lyon Neuroscience Research Center (CRNL), Lyon, France; 5 University of Lyon 1, Lyon, France; 6 CERMEP-Imaging Platform, Bron, France; 7 SFR Lyon-Est, Université Lyon 1, CNRS UAR3453, INSERM US7, Lyon, France; 8 Department of Psychology, National University of Singapore, Singapore, Singapore; 9 Cécile & Oskar Vogt Institute of Brain Research, Medical Faculty and University Hospital Düsseldorf, Heinrich Heine University, Düsseldorf, Germany

**Keywords:** Cognitive neuroscience, Neural circuits

## Abstract

Current theories fail to explain why the ability to control speech is unique to humans. We recently identified one unique feature in the human frontal cortex that may hold the key to this question: the Prefrontal Operculum (PFO). Here we aim to identify 1) its anatomo-functional organization to elucidate its potential function and 2) whether it has a homolog in the macaque brain. Functional connectivity (FC) results in humans, revealed that PFO is subdivided in two regions (aPFO and pPFO), displaying strong interactions but distinct whole brain FC profiles with respectively the language and the cognitive control networks, and thus suggesting an important role of PFO in the cognitive control of speech. Connectivity fingerprint analyses in macaques revealed similarities with pPFO, but we found no macaque homolog of human aPFO. Altogether, this study points toward the emergence of aPFO as an evolutionary advantage in hominids for modern speech abilities.

## Abstract (web summary)

Human and macaque frontal operculum connectivity patterns point to a uniquely human subdivision in the prefrontal cortex that may serve as a control hub for some speech functions.

## Introduction

**Fig. 1:**
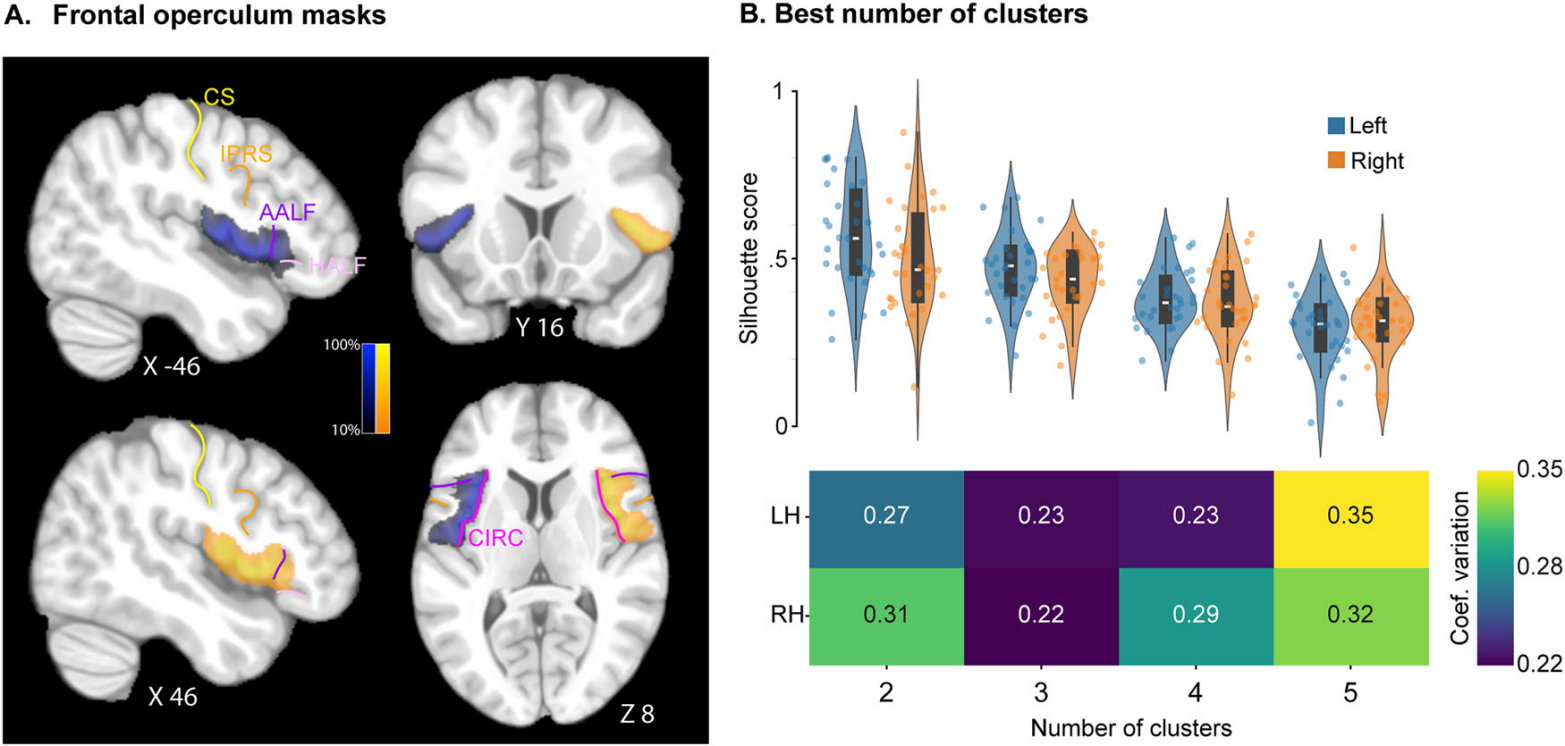
Probability map of the frontal operculum mask in the left and right hemispheres across all participants and identification of the best number of subdivisions of the masks. **A** The probability maps of the masks are shown on sagittal, coronal, and axial sections of the non-linear MNI template. The color bars represent the % of subjects displaying the frontal operculum mask in a given voxel. The probabilistic maps of the left (orange) and right (blue) frontal operculum is provided in Supplemental Data 1. **B** The best number of subdivisions was identified by extracting the correlation (*Z*-value) between the BOLD signal in the low frequency band (0.01–0.1 Hz) observed in each voxel of the mask and all voxels of the whole-brain gray matter segmentation mask. Next, cross-correlation is computed to the latter to obtain a correlation-based distance between voxels of the mask. Finally, applying spectral clustering on the resulting matrix. The best number of subdivisions corresponds to the number associated with the highest value of Silhouette score for a given subject (**B**, top diagram). The group level analysis accounts for the variability of the Silhouette score, and the number of clusters associated with the lowest coefficient of variation is the optimal one (**B**, bottom diagram). Results show that the best number of subdivisions of the frontal operculum mask in both hemispheres is 3. Note that, in (**B**), white bars correspond to the median of group distributions, the surrounding rectangles represent the interquartile range (IQR = 25th–75th percentiles), the whiskers extend to the most extreme values within 1.5 × IQR from the lower and upper quartiles, and individual dots are single observations. IPRS inferior precentral sulcus, CS central sulcus, AALF ascending anterior ramus of the lateral fissure, HALF horizontal anterior ramus of the lateral fissure, CIRC circular sulcus.

The evolutionary origins of human speech remain one of the most mysterious research questions^1^ as the prominent theories (i.e., position of the larynx, neurogenetic adaptations) have been rejected^2,3^. In addition, most of the neural infrastructure for speech in the frontal cortex was thought to be already present in non-human primates^4^, albeit at a nascent stage, including the classical Broca’s area (i.e., area 44)^5,6^. However, we recently identified one unique feature of adult human brain anatomy that might provide the missing piece to this puzzle^7,8^. This region, located adjacent and medial to the classical Broca’s area, is called the PreFrontal extent of the frontal Operculum (PFO), and is part of the larger frontal operculum which extends posteriorly to the central sulcus (*CS*, Fig. 1A). The goal of the present paper is to identify what makes this region human-specific by assessing its anatomo-functional organization based on its whole-brain connectivity pattern in humans and in macaques.

During cortical development in primates, the lateral frontal cortex folds over the insula, which is dorsally bordered by the circular sulcus (*CIRC*). The buried part lateral to the insula corresponds to the frontal operculum. Whereas this entire region remains at the level of the precentral gyrus and thus of the premotor/motor cortex in non-human primates (PCO, PreCentral Operculum)^8^, it also comprises an additional -prefrontal cortical- part in humans (PFO, PreFrontal Operculum, Fig. 1). This reflects the expansion of the prefrontal cortex (particularly areas 9 and 10^7,9^) which is associated with a posterior displacement of the anatomical boundary between the prefrontal and premotor cortices, marked by the inferior precentral sulcus^7,8^ in humans^10^. Contrary to non-human primates, the anterior part of the insula is consequently located at the level of the prefrontal cortex in humans, and is thus covered by prefrontal cortical areas, i.e., by PFO. PFO is thought to belong to the larger Broca’s complex, which also contains the ventrolateral prefrontal cortical areas 44 in the pars opercularis and 45 in the pars triangularis (Fig. 1)^11^. At this level, the ascending anterior ramus of the lateral fissure (*AALF*, the rostral border of the classical Broca’s area 44) is convoluted to the point that it joins the *CIRC*, creating a complete opercularization (Fig. 1). This complete opercularization is of utmost importance because the horizontal ascending ramus of the lateral fissure (*HALF*) emerges from the *AALF/CIRC* junction (Fig. 1), thereby creating the fold that defines the pars triangularis. By contrast, old-world monkeys (including baboons and macaques) anatomically lack PFO as the frontal operculum remains exclusively at the level of the premotor/motor cortex, and chimpanzees lack a complete opercularization of the PFO. As such, all studied non-human primates (i.e., macaques, baboons, chimpanzees, humans) lack a complete Broca’s complex^8^. In contrast, the sulcal organization of the other frontal cortical regions, both medial and lateral, can be found in the aforementioned non-human primate species^7,9^. Although these latter studies strongly point toward the anatomical human uniqueness of PFO, it has been neglected so far, and its anatomo-functional organization remains largely misunderstood.

The primary goal of this study was to identify the anatomo-functional organization and thus the potential function^12^ of PFO in the human brain by assessing the whole-brain functional connectivity (FC) of the entire frontal operculum (including PFO and PCO) thanks to resting-state functional Magnetic Resonance Imaging (rsfMRI), a method based on spontaneous low-frequency fluctuations of blood-oxygenation-level-dependent (BOLD) signal (<0.1 Hz) at rest^13^. Indeed, a given area displaying a specific laminar organization (cytoarchitectonic organization) is associated with a particular connectivity that constrains its function^12^. However, assessing the specific function of PFO with voxel-based neuroimaging techniques warrants a meticulous approach. The spatial configuration of the region makes it extremely challenging to separate activity located in PFO from the anterior insula^14^, and from the lateral adjacent areas 44 and 45, particularly when using group analysis of functional magnetic resonance imaging (fMRI) data, which is the most common methodology worldwide. The present paper overcomes this challenge by setting the sulcus inter-individual morphological variability at the center of all analyses. Indeed, the organization of sulci in the human brain provides unique landmarks for the anatomo-functional organization, and using such landmarks has demonstrated its value by solving major issues related to the understanding of the anatomo-functional organization of various parts of the brain ^(e.g.,^ ^6,14–22^^)^. The secondary goal was to take advantage of our database on 18 anesthetized macaques to attempt to identify whether, although not anatomically present, a functional homolog of PFO could be identified in the macaque brain using fingerprint connectivity matching methods^23–25^.

The results reveal that the human frontal operculum is subdivided into 3 regions, organized on the antero-posterior axis: PCO, located at the level of the precentral gyrus and thus the premotor/motor cortex, and the posterior (pPFO) and anterior (aPFO) prefrontal operculum, both located in the prefrontal cortex. These regions display highly specialized FC patterns with the entire brain that strongly suggest an important role of the left PFO in the cognitive control of some aspects of speech. Of utmost importance, aPFO is the only region that does not find a functional homolog in the macaque brain, thus emphasizing that the appearance of this region may have accompanied the emergence of speech.

## Results

### The frontal operculum in humans

To identify the anatomo-functional organization of the frontal operculum based on its FC profile in humans, the strategy consisted of three main steps: the *first step* was to identify and manually delineate a mask covering the frontal operculum based on structural MRI scan of each participant and each hemisphere; the *second step* was to use a data-driven clustering method, spectral clustering, to parcellate and identify the optimal number of subregions within the frontal operculum mask for each individual participants and each hemisphere (see “Methods”, and refs. ^25^^,^^7^); the *third step* was to assess the whole-brain FC profiles of each of the resulting subregions.

#### Frontal operculum mask and relation with anatomy

The frontal operculum (including its premotor/motor and prefrontal parts) was first visually and manually labeled in each hemisphere: the central sulcus (*CS*) was the caudal limit of the mask, the rostral end of the insula was the rostral limit, the fundus of the circular sulcus (*CIRC*) was the medial limit, and laterally, the gray matter of the lateral adjacent areas located on the surface was excluded. The average volume of the frontal operculum mask was similar in the left and right hemispheres (left hemisphere: average ± stdev = 5142.7 ± 1003 mm^3^, right hemisphere: average ± stdev = 5104.9 ± 643 mm^3^; General Mixed Linear Model—GLMM, see “Methods” : *F* = 0.0092, NumDF = 1, DenDF = 184, *p* = 0.9236 (ns), Fig. 1A).

#### Frontal operculum parcellation and macroscale local anatomy

Based on the whole-brain FC pattern of each voxel within the frontal operculum mask in each hemisphere and using data-driven spectral clustering (Fig. 1, see Methods—Data-driven parcellation), we identified the best number of subregions composing the mask by evaluating Silhouette score measures and their variances across individuals. Specifically, we employed the coefficient of variation to identify the number of subregions that showed the greatest consistency across our group of participants. The frontal operculum was best parcellated in 3 subregions, in both hemispheres (score in the left hemisphere = 0.23, score in the right hemisphere = 0.22 for 3 parcels, Fig. 1B).

**Fig. 2:**
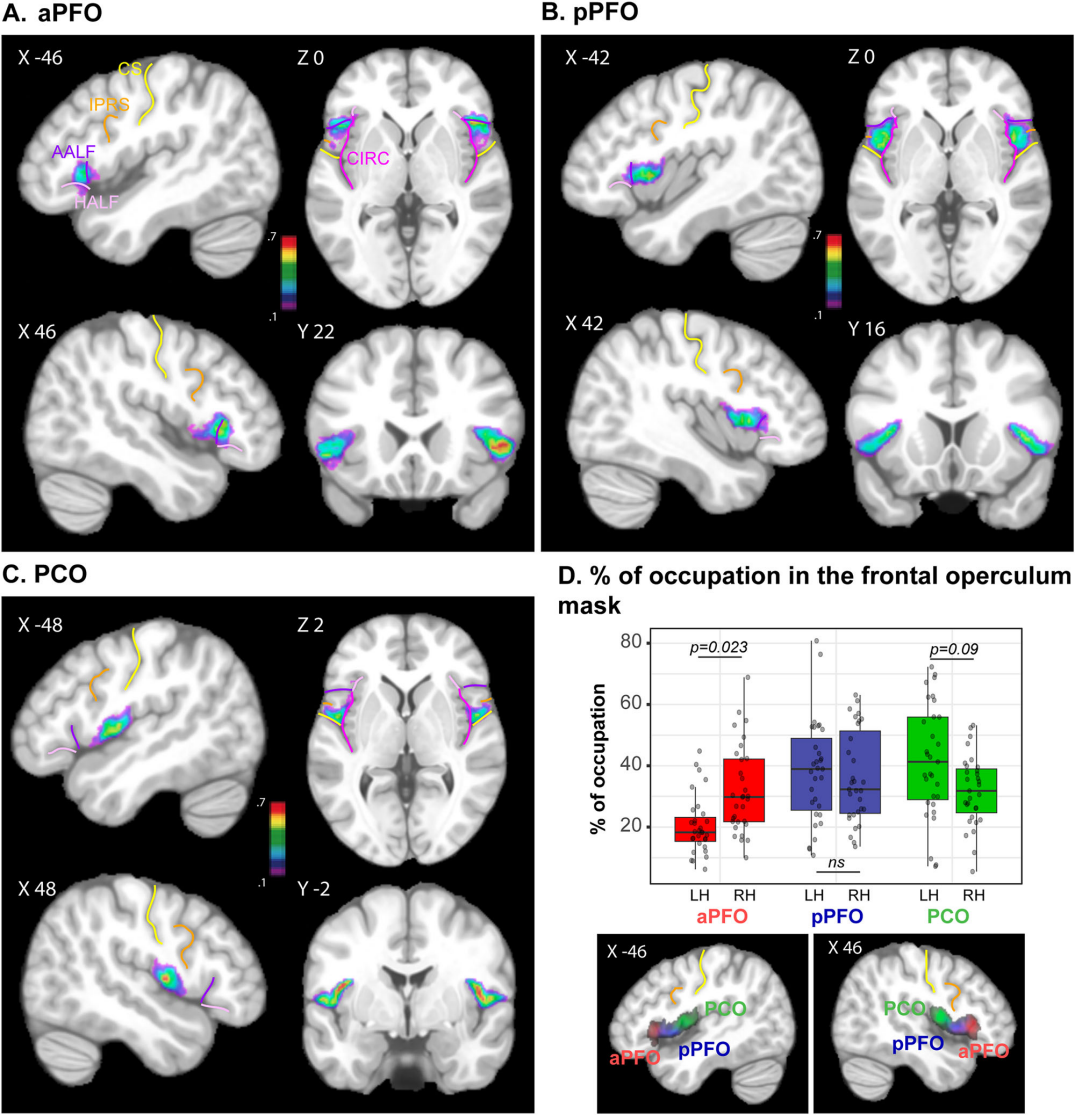
Probability maps of each frontal operculum subregions and their percent of occupations in all participants in both hemispheres. Probabilistic maps of aPFO (**A**), pPFO (**B**), and PCO (**C** are presented on the asymmetric non-linear ICBM 152 MNI anatomical template. *X*, *Y*, and *Z*-values represent, respectively, the medio-lateral, antero-posterior, and dorsoventral levels of the presented slices in the standard MNI space. The color scale represents the probability to find a given region (aPFO, pPFO, or PCO) across all participants. The probabilistic maps of each region in 3D are provided in Supplemental Data 2. **D** % of occupation of aPFO, pPFO, and PCO in the left versus right hemisphere. The location and the % of occupation of aPFO, pPFO, and PCO are presented in red, blue, and green, respectively. Post hoc Tukey tests reveal that aPFO occupies less volume in the left than in the right hemisphere, whereas PCO in the left tends to occupy more volume in the left than in the right hemisphere. Source data are provided in Supplemental Data 3). Note that, in (**D**), horizontal bars correspond to the median of the distributions, the surrounding rectangles represent the interquartile range (IQR = 25th–75th percentiles), the whiskers show the range of values within 1.5 × IQR from the lower and upper quartiles, and individual dots are single observations. CIRC, circular sulcus, CS, central sulcus, IPRS, inferior precentral sulcus, AALF and HALF, ascending and horizontal ramus of the anterior lateral fissure, LH and RH, left and right hemisphere.

These 3 subregions were consistently organized along a caudo-rostral axis in all individuals (Figs. S1 and S2), as reflected in the probabilistic map of these regions across participants (Fig. 2). This analysis revealed that the posterior part (PCO) is located between the central sulcus (*CS*) and the inferior precentral sulcus (*IPRS*) and thus at the level of the motor/premotor cortex (Fig. 2C). Both the posterior and anterior PFO (pPFO and aPFO) are located in front of the inferior precentral sulcus and thus lie in the prefrontal cortex^7^. Specifically, pPFO is located between the *IPRS* and *AALF* and is thus at the level of the pars opercularis of Broca’s area (Fig. 2B). The aPFO is located between *HALF* and *AALF* and thus at the level of the pars triangularis of Broca’s area (Fig. 2A).

In addition, results revealed a differential occupation of the 3 subregions in the left versus right hemispheres within the frontal operculum region (i.e., as measured by the volume occupied by each subregion/total volume of the frontal operculum * 100). First, whereas the 3 subregions occupied proportionally the same volume in the right hemisphere (ns at *p* < 0.05), aPFO occupied less volume compared to both pPFO (*p* < 0.0001) and PCO in the left hemisphere (pPFO vs PCO: non-significant at *p* < 0.05). Second, aPFO was smaller and PCO larger in the left versus right hemisphere, while pPFO was of similar volume in both left and right hemispheres (as captured by the interaction “subregion * hemisphere”: *F* = 8.53, NumDF = 2, DenDF = 120, *p* = 5.081e–06, GLMM). Marginal *R*^2^ shows that fixed effects explain 25.1% of the variance, which is common in complex biological data^26^.

#### Left hemispheric ipsilateral whole-brain functional connectivity

**Fig. 3:**
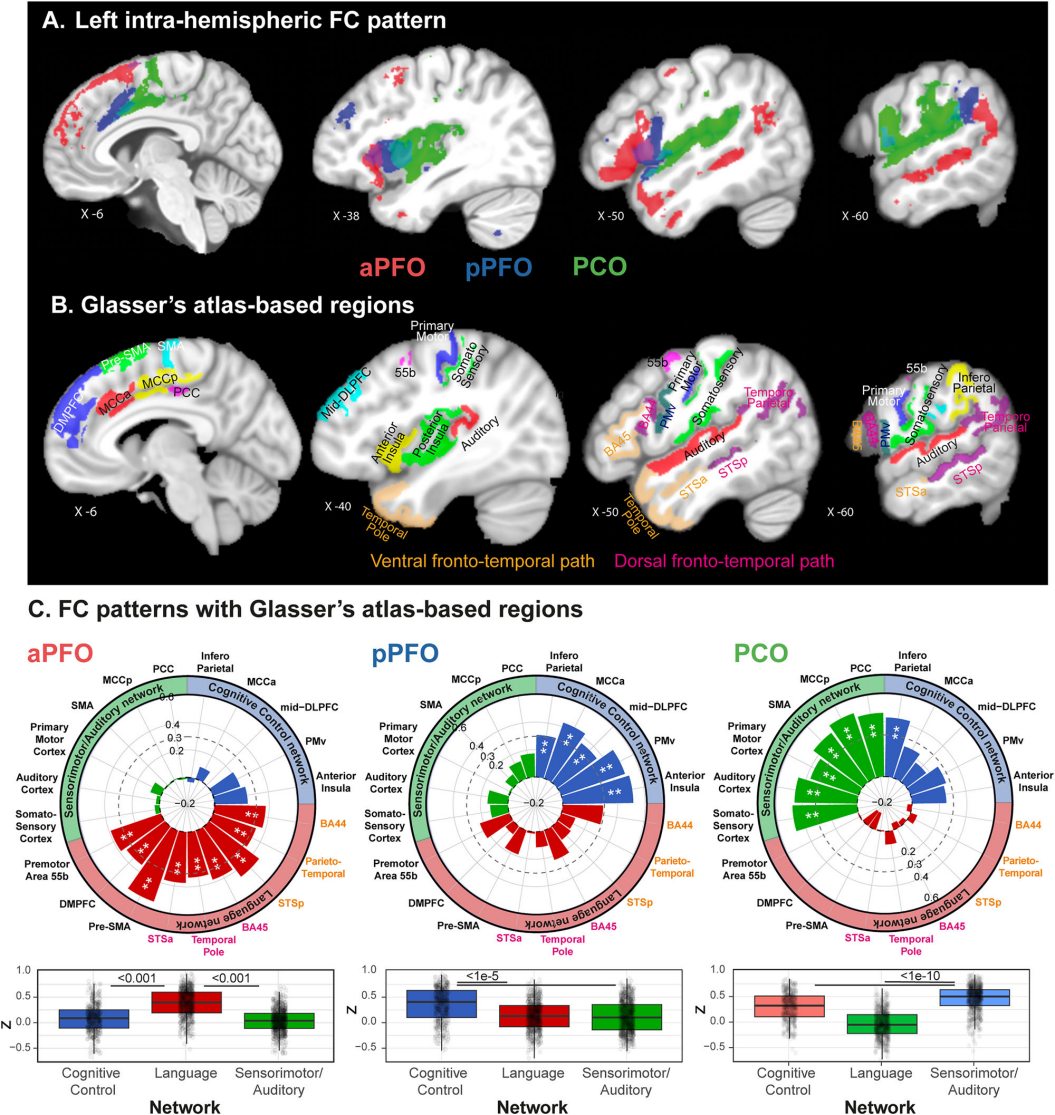
Left ipsilateral FC patterns of aPFO (in red), pPFO (in blue), and PCO (in green). **A** FC patterns are displayed on the non-linear anatomical MNI template. *X* values represent the medio-lateral level of the sagittal slice. The whole-brain unthresholded FC maps of each region in 3D is provided in Supplemental Data 4. **B** Corresponding regions from the Glasser’s atlas (see Table 1). Note that the regions belonging to the dorsal and ventral fronto-temporal language paths are presented, respectively, in dark pink and orange. **C** Average correlation (*Z* values) across 31 participants between the BOLD in each frontal operculum region and each region defined in **B**) (spider plots) and each network (boxplot) (source data are provided in Supplemental Data 5). Note that, in (**C**), horizontal bars correspond to the median of the distributions, the surrounding rectangles represent the interquartile range (IQR = 25th–75th percentiles), the whiskers show the range of values within 1.5 × IQR from the lower and upper quartiles, and individual dots are single observations. In spider plots, **represents a significant FC pattern at *p* < 0.001, the dotted line representing the threshold at *p* < 0.01 (*Z* = 0.3). In the boxplots are presented significant post-hoc tests from GLMM (see “Methods”).

The 3 subregions display distinct FC patterns with the entire cortex of the ipsilateral hemisphere (Fig. 3A). Specifically, aPFO displays significant FC (in red, Fig. 3A) with the medial prefrontal cortex (including pre-SMA and the dorsomedial prefrontal cortex -DMPFC-), the anterior insula, the lateral premotor area 55b, with the dorsal language pathway (including BA 44, the parieto-temporal intersection and more specifically Wernicke’s area and the temporo-parietal junction (TPJ), as well as the superior temporal sulcal cortex located posterior to the sulcus acousticus), and the ventral language pathway (including BA 45, the temporal pole and the superior temporal sulcal cortex located anterior to the sulcus acousticus). By contrast, pPFO displays significant FC (in blue, Fig. 3A) with the anterior midcingulate cortex (MCCa), the mid-dorsolateral prefrontal cortex (mid-DLPFC), the ventral premotor cortex (PMv), and the infero-parietal cortex. Finally, PCO displays significant FC with the sensorimotor-auditory cortex, including the primary and secondary auditory cortices, the somatosensory cortex, the primary motor cortex, and the medial premotor cortex. Note that these three networks are displayed in detail in Fig. S3. Based on the literature, the regions described above can be classified into 3 types, depending on the network they belong to: those belonging to the language, cognitive control, and the sensorimotor-auditory networks (see Table 1 for details on the classification and associated references). These results point to a high functional specialization of these subregions with aPFO, pPFO, and PCO, respectively belonging to the language, cognitive control, and sensorimotor-auditory networks (Fig. 3A, see Table 1).

**Table 1:** Composition of the regions assessed in the present study and the Glasser’s atlas ROIs that they encompass in the left (LH) and right (RH) hemispheres

Regions	Network	Glasser’s atlas ROIs in LH^27^	Glasser’s atlas ROIs in RH
***Medial surface***			
DMPFC	Language^85^	8Bl, 9 m, d32	8Bl, 9 m, d32
MCCa	Cognitive Control^16^	a24pr, p32pr, a32pr	a24pr, p32pr, a32pr, SCEF
MCCp	Sensorimotor-Auditory^18^	p24pr, 24dd, 24dv	p24pr, 24dd, 24dv
PCC	Sensorimotor-Auditory^18^	23c, 23 d	23c, 23 d
Pre-SMA	Language^86^	SFL	SFL
SMA	Sensorimotor-Auditory^86^	6 mp	6 mp
***Lateral surface***			
Mid-DLPFC	Cognitive Control^87,88^	46, 9–46 d	46, 9–46 d
BA 44	Language^11,89^	44, IFJa	44, IFJa
BA 45	Language^11,89^	45, IFSa, IFSp	45, IFSa, IFSp
Premotor area 55b	Language^90^	55b	55b
Ventral premotor cortex	Cognitive Control^91^	6r	6r, 6 v
Primary motor cortex	Sensorimotor-Auditory^92^	4	4
Infero-parietal cortex	Cognitive Control^93^	PF, PFop	PF, PFop, PFm
Temporo-parietal cortex	Language^94^	STV, PSL, PGi	STV, PSL, PGi
STSp	Language^95^	STSvp, STSdp	STSvp, STSdp
STSa	Language^95^	STSva, STSda	STSva, STSda
Somatosensory cortex	Sensorimotor-Auditory^96^	Primary: 1, 3a, 3b Secondary: OP1, OP2-3, OP4, 43, PFcm + posterior insula (see below)	Primary: 1, 3a, 3b Secondary: OP1, OP2-3, OP4, 43, PFcm + posterior insula (see below)
Auditory cortex	Sensorimotor-Auditory^97^	Primary: A1, LBelt, MBelt, PBelt, RI Secondary: TA2, A4	Primary: A1, LBelt, MBelt, PBelt, RI Secondary: TA2, A4
Temporal pole	Language^98^	STGa, TGd, TGv	STGa, TGd, TGv
***Insula***			
Anterior insula	Cognitive Control^71^	AAIC, AVI, MI	AAIC, AVI, MI
Posterior insula	Sensorimotor-Auditory^99^	Ig, Pol1, Pol2, 52	Ig, Pol1, Pol2, 52

*DMPFC* dorsomedial prefrontal cortex, *MCC*a and *MCC*p anterior and posterior mid-cingulate cortex, *PCC* posterior cingulate cortex, *Pre-SMA* pre-supplementary motor area, *SMA* supplementary motor area, *mid-DLPFC* mid-dorsolateral prefrontal cortex, *STSp* and *STSa* posterior and anterior part of the superior temporal sulcus.

We then identified, in the Glasser’s atlas^27^, which discrete regions of interests (ROIs) corresponded to the various nodes of the 3 networks described above (Fig. 3A). Some nodes corresponded to only one Glasser’s atlas subdivision (e.g., Primary motor cortex = Glasser’s area 4) but some nodes overlapped with more than one Glasser’s atlas subdivision (e.g., Area 46 and 9–46 d). In this latter case, we combined them in a new ROI (e.g., Mid-DLPFC includes both areas 46 and 9–46, see literature in supplemental data of Glasser et al.^27^). These newly defined ROIs are presented in Fig. 3B and described in detail in Table 1. We then measured FC between each frontal operculum subregion and these ROIs (see Methods and Fig. 3A).

Results (Fig. 3C) confirm that aPFO, pPFO, and PCO display significant FC (at *p* < 0.01, *Z* > 0.3) with all regions identified as belonging to the language (*F* = 51.748, NumDF = 1, DenDF = 30, *p* < 1.883e–10, GLMM, Marginal *R*^2^ = 0.374), cognitive control (*F* = 38.215, NumDF = 1, DenDF = 30, *p* < 5.634e–9, GLMM, Marginal *R*^2^ = 0.141), and sensorimotor-auditory (*F* = 118.49, NumDF = 1, DenDF = 30, *p* < 4.183e–15, GLMM, Marginal *R*^2^ = 0.514) network, respectively. Note that marginal *R*^2^ shows that fixed effects explain 37.4% (*R*^2^ = 0.374), 14.1% (*R*^2^ = 0.141), and 51.3% (*R*^2^ = 0.513) of the variance, respectively, for FC patterns of aPFO, pPFO, and PCO in the left hemisphere.

#### Right hemispheric ipsilateral whole-brain Functional connectivity

**Fig. 4:**
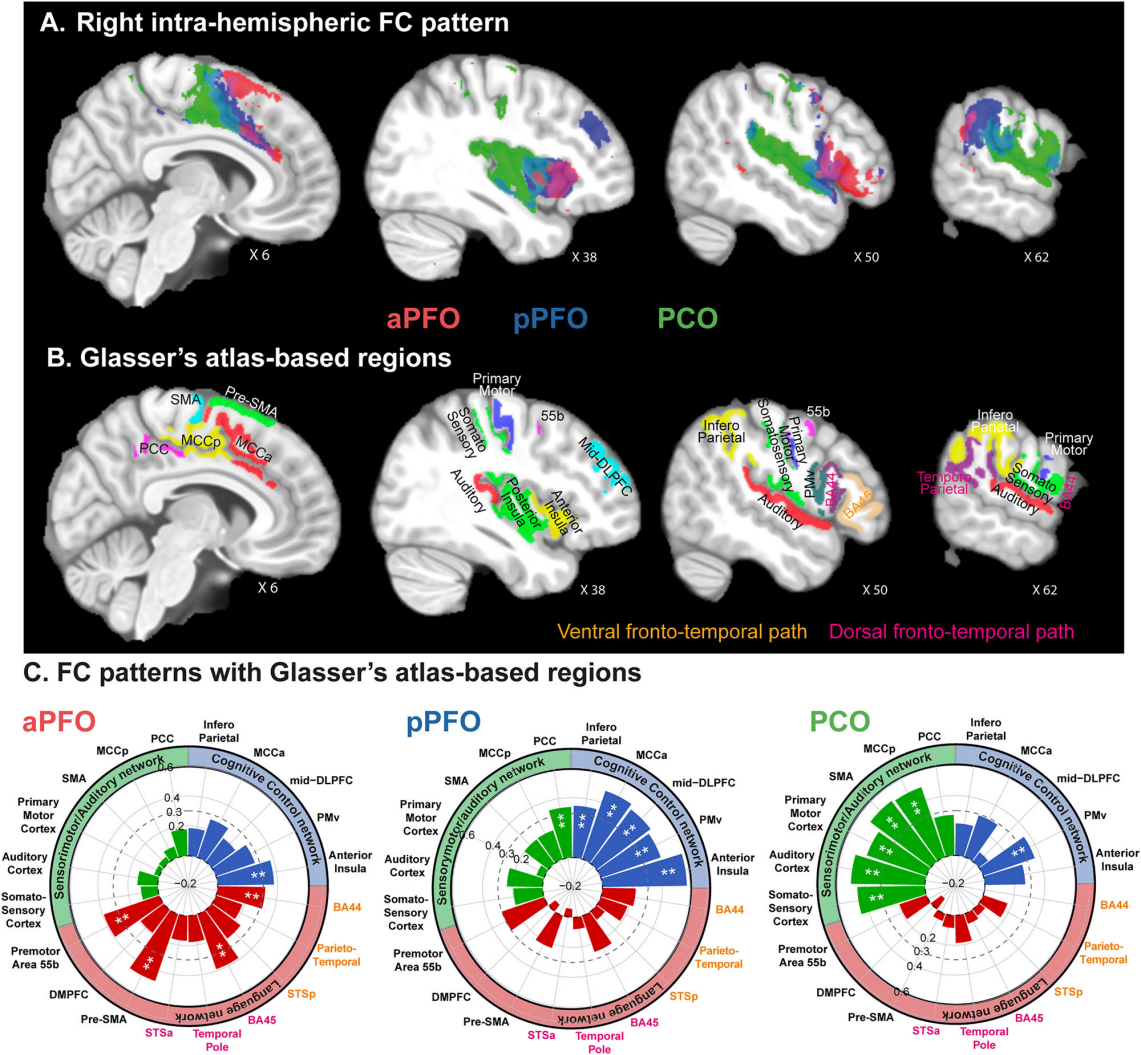
Right ipsilateral FC patterns of aPFO (in red), pPFO (in blue), and PCO (in green). **A** FC patterns are displayed on the non-linear anatomical MNI template. *X* values represent the medio-lateral level of the sagittal slice. The whole-brain unthresholded FC maps of each region in 3D are provided in Supplemental Data 4. **B** Corresponding regions from Glasser’s atlas (see Table 1). Note that the regions belonging to the dorsal and ventral fronto-temporal language path are presented, respectively, in dark pink and orange. **C** Average correlation (*Z*-values) across 31 participants between the BOLD in each frontal operculum region and each region defined in (**B**) (spider plots). Note that source data are provided in Supplemental Data 5. In spider plots, **represents a significant FC pattern at *p* < 0.001, the dotted line representing the threshold at *p* < 0.01 (*Z* = 0.3).

Figure 4A shows that the 3 frontal operculum subregions display distinct FC patterns. In the right hemisphere, pPFO and PCO subregions present similar FC patterns to those observed in the left hemisphere. Specifically, as in the left hemisphere, pPFO displays significant FC (in blue, Fig. 4A) with the anterior MCCa, the mid-dorsolateral prefrontal cortex (mid-DLPFC), the ventral premotor cortex (PMv), the anterior insula, and the infero-parietal cortex. Similarly, PCO displays significant FC with the sensorimotor-auditory cortex, including the primary and secondary auditory cortices, the somatosensory cortex, the primary motor cortex, and the medial premotor cortex. Note that these three networks are displayed in detail in Fig. S4. These results thus show that pPFO and PCO belong, respectively, to the cognitive control and sensorimotor-auditory networks in both the left and right hemispheres. By contrast, the FC pattern of aPFO is different from that in the left hemisphere. aPFO in the right hemisphere displays FC with the preSMA, anterior insula, lateral premotor area 55b, lateral prefrontal BA 44 and 45 (in red, Fig. 4A). Importantly, the main difference between the FC pattern of aPFO in the right compared to the left hemisphere lies in the connectivity with the temporo-parietal and temporal cortex that is clearly absent in the right hemisphere and present in the left hemisphere.

As in the left hemisphere, we identified in Glasser’s atlas the ROIs concerned by these aforementioned networks and extracted the correlation *Z*-values between each frontal operculum subregion and the newly defined regions (displayed in Fig. 4B and Table 1). Results are presented in Fig. 4C and confirm that pPFO and PCO display significant FC (at *p* < 0.01, *Z* > 0.3) with all regions identified as belonging to the cognitive control and sensorimotor-auditory network, respectively. By contrast, aPFO displays significant FC with a local ventrolateral prefrontal–ventral premotor network.

#### Functional connectivity between aPFO, pPFO, and PCO

**Fig. 5:**
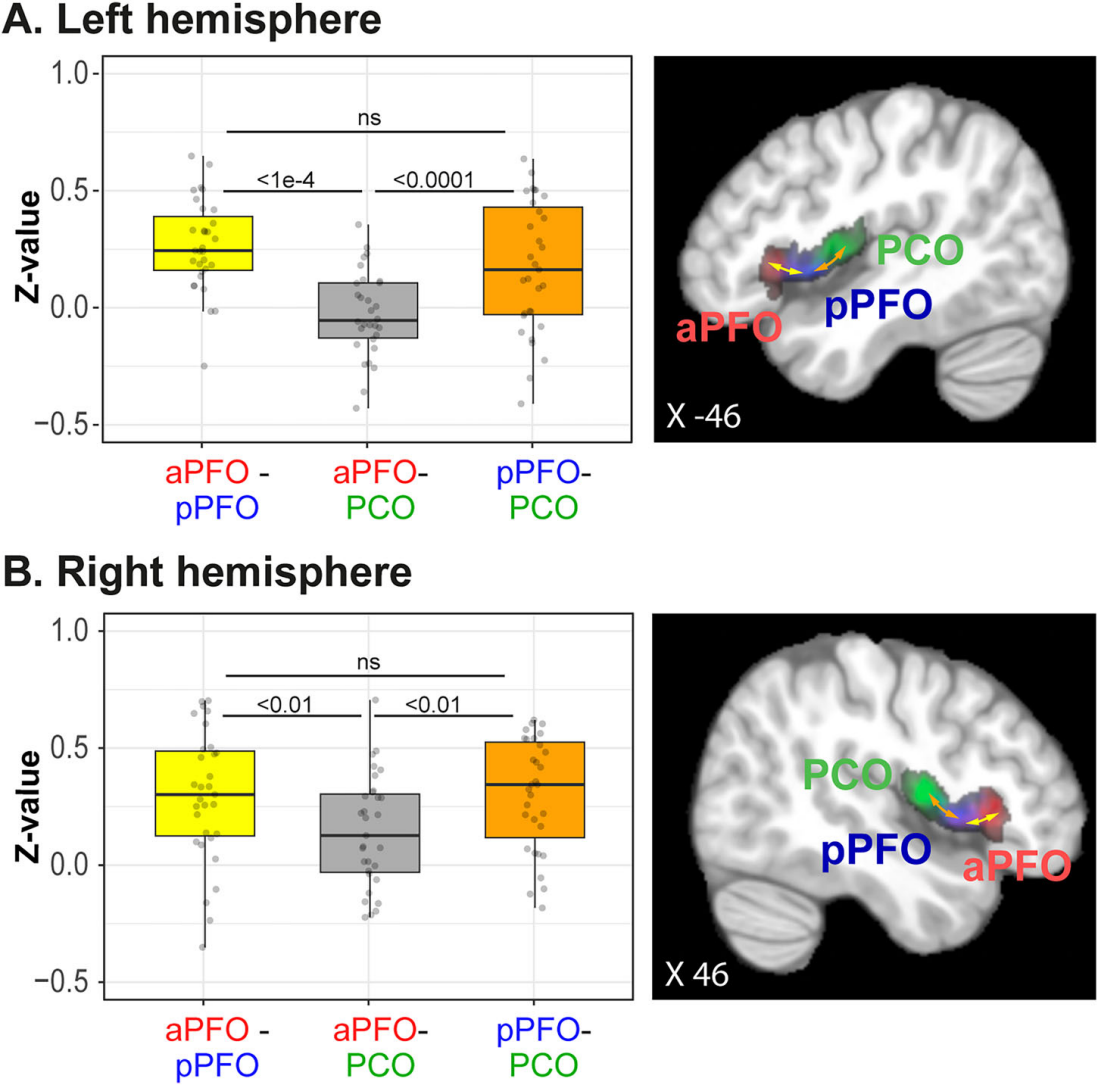
Functional connectivity between aPFO, pPFO, and PCO. Boxplots represent the distribution of *Z*-values in the left (**A**) and right (**B**) hemispheres across 31 participants between aPFO and pPFO (in yellow), pPFO and PCO (in orange), and aPFO and PCO (in gray). Results show increased FC between pPFO and aPFO (see also the yellow arrow on the sagittal view displaying the probability maps of the three frontal operculum subregions) and between pPFO and PCO (see orange arrow), suggesting a coordinating role of pPFO in both hemispheres. Source data are provided in Supplemental Data 6. Note that, in boxplots, horizontal bars correspond to the median of the distributions, the surrounding rectangles represent the interquartile range (IQR = 25th–75th percentiles), the whiskers show the range of values within 1.5 × IQR from the lower and upper quartiles, and individual dots are single observations. ns non-significant.

We then assessed the local FC between aPFO, pPFO, and PCO in both hemispheres. Figure 5 shows that pPFO displays increased FC with both aPFO and PCO, whereas these two latter regions do not appear to be significantly functionally connected with each other (left hemisphere: *F* = 18.232, NumDF = 2, DenDF = 60, *p* < 6.512e-7; right hemisphere: *F* = 5.161, NumDF = 2, DenDF = 60, *p* < 0.085, GLMM). These results strongly suggest that bilaterally, pPFO interfaces the three networks originating from each frontal operculum subregion.

### The frontal operculum in macaques

The analysis of the sulcal pattern of the frontal cortex across primates strongly suggests that PFO is anatomically human-specific. In humans, it corresponds to the portion covering the anterior insula at the level of the prefrontal cortex, whereas in non-human primates, the anterior insula remains posterior to the prefrontal cortex^7^. A critical unsolved question is thus the following: *do non-human primates have a functional homolog of the human PFO?* To address this question, we adopted the connectivity fingerprint matching approach^25^ to look for homologous regions in the macaque brain with the same FC profile as the human aPFO and pPFO regions. Note that the homolog of the human PCO in the macaque brain is already known: it is located in the buried part adjacent to the insula at the level of the premotor/motor cortex and corresponds to area PrCO^28^. The key idea behind this approach is that a particular brain area can be defined based on its unique pattern of connections^29^, and this unique connectivity can therefore serve as a fingerprint for identifying the same area across different brains across individuals and species^23,25^.

**Fig. 6:**
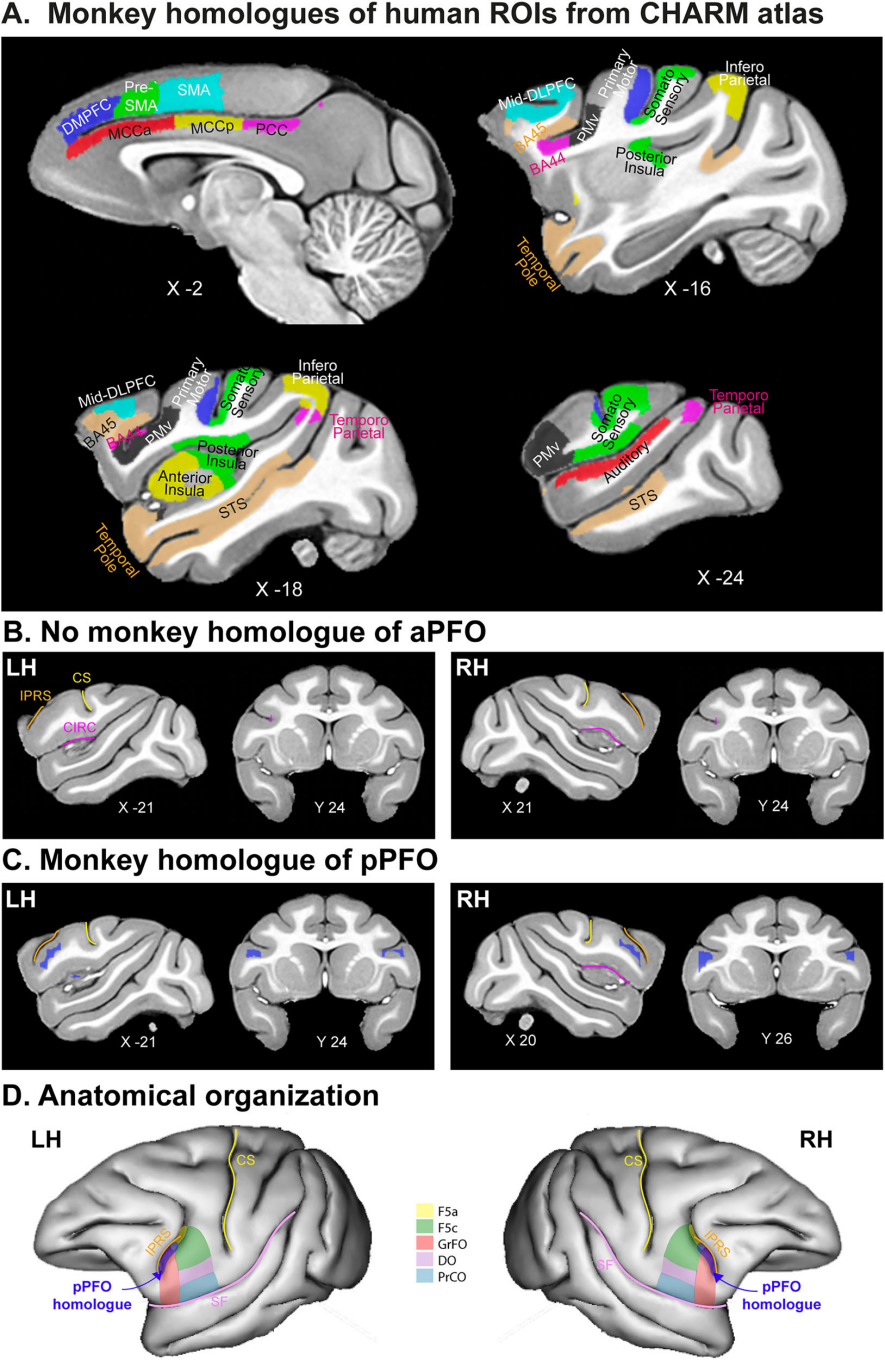
Location of the macaque functional homologs of the human aPFO and pPFO in both hemispheres, based on their FC matching profiles with the homologs. **A** Macaque homologs of the human ROIs from spider plots in Fig. 3 (Targets). Results obtained from 18 monkeys show that aPFO does not find a functional homolog in the macaque brain (**B**), whereas pPFO finds one (**C**), both in the left (LH) and right (RH) hemispheres (at *p* < 0.05 FWE corrected). **D** From the cytoarchitectonic map of Gerbella et al.^31^, the macaque homolog of the human pPFO (in dark blue) lies mainly in area GrFO in both hemispheres. Results are shown on the CHARM anatomical template. *X* and *Y* values represent, respectively, the medio-lateral and antero-posterior level of the slice in the CHARM standard space.

The first step was to identify in macaques the set of *target* brain regions, i.e., the anatomical homologs of the human ROIs found to be connected to the aPFO, pPFO, and PCO from our FC analyses (Fig. 3C). These ROIs were identified in the macaque monkey from the macaque Charm (level 6) atlas (see Methods). Note that in this atlas, the ROI STS does not separate the anterior from the posterior portion; thus, whereas we have two STS ROIs in humans (i.e., STSa and STSp), we have only one in macaque (i.e., STS). As aPFO is equally functionally connected with both STSa and STSp (see Fig. 3C), we did not reasonably expect that it would impact the results. Note also that the existence of a macaque homolog for the human premotor area 55b remains uncertain; consequently, this was the only region of interest excluded from our analysis. Thus, whereas 20 ROIs were described in humans, 18 were identified in macaques and used for the analysis. These ROIs are described in Table 2 and shown in Fig. 6A. Then, using our dataset of resting-state fMRI data obtained in 18 anesthetized macaques^30^, we sought to identify the voxels in the macaque brain that have similar FC pattern with the homologs of the human target regions as do aPFO and pPFO in humans (see Methods section for more details about the connectivity fingerprint matching method). As shown in Fig. 6D, the connectivity matching fingerprint analysis revealed no voxels in the macaque brain that showed a similar FC profile as the human aPFO, both in the left and right hemispheres (Fig. 6B). This result suggests that the homolog of the human aPFO might not be present in the macaque brain. Nonetheless, this analysis revealed voxels in the macaque brain that show a similar FC profile as the human pPFO. These voxels were located in area GrFO and the arcuate sulcal part of area FS (i.e., F5a)^31^ and suggested that these regions may be functionally homologs of the human pPFO regions (Fig. 6C).

**Table 2:** Composition of the regions from the CHARM atlas corresponding to the anatomical homologs of the human ROIs identified in Fig. 3

Regions	CHARM atlas ROIs in LH
***Medial surface***	
DMPFC	8Bm, 9 m
MCCa	24c
MCCp	24c’
PCC	23c
Pre-SMA	Pre-SMA
SMA	SMA
***Lateral surface***	
Mid-DLPFC	Areas 46 d, 46 v, 46 f
BA 44	Area 44
BA 45	Area 45
Ventral premotor cortex	F5
Primary motor cortex	M1
Infero-parietal cortex	7a
Temporo-parietal cortex	Tpt
STS	FST, TEa, PGa, IPa, TEm, TPO
Somatosensory cortex	1-2, 3a, SII + Posterior insula
Auditory cortex	RPB, RTM, ML, AL, RTL
Temporal pole	TGdd, TGvd, TGdg, TGvg, TGsts, TGa
***Insula***	
Anterior insula	Ia-IdI
Posterior insula	Ig

## Discussion

The present paper provides a fine-grained anatomo-functional organization of the frontal operculum in the human brain based on local macroscale sulcal organization and identifies to what extent it is human-specific. First, results reveal that, in humans, this region does possess three subregions organized rostro-caudally, two being located in the prefrontal cortex (aPFO and pPFO are located at the level of the pars opercularis and pars triangularis of the ventrolateral prefrontal cortex, respectively) and one in the premotor/motor cortex PCO. In the left hemisphere, aPFO, pPFO, and PCO display FC with, respectively, the language, cognitive control, and sensorimotor-auditory networks, with pPFO that potentially interfaces the three networks as it significantly correlates with both aPFO and PCO. This is a specificity of the left hemisphere because the FC pattern of aPFO in the right hemisphere lacks connectivity with the temporal cortex and thus with the fronto-temporal language network. Second, results reveal that aPFO does not find a functional homolog in macaques as they do not present any brain region with a FC pattern similar to the human aPFO (see Fig. 6C). Combined with additional comparative neuroscience^7–9^ and neuroarcheological evidences^32,33^, these results are supporting the hypothesis that the emergence of aPFO may have conferred an evolutionary advantage for supporting complex manipulation of cognitive representations required for some critical aspects of speech and language processing.

The data-driven parcellation of the frontal operculum along a rostro-caudal axis, derived from the whole-brain FC patterns of its voxels, aligns well with its cytoarchitectonic structure. Specifically, PCO, pPFO, and aPFO appear to correspond to areas Op 5–6, Op 7–8, and Op 9–10, respectively^11,34^. However, we did not capture the fine-grained medio-lateral organization (i.e., Op5 versus Op6, Op7 versus Op8, and Op9 versus Op10). This might be due to methodological limitations (i.e., spatial resolution of the MRI data) or to the possibility that two frontal opercular areas on this medio-lateral organization do not display main differences in their whole-brain FC patterns. Such assessments would require further investigations using fMRI data with higher spatial resolution. Results show that aPFO is smaller than the other subregions in the left hemisphere, but this is not the case in the right hemisphere. Although it remains unclear whether the size of a cortical area reflects its level of specialization, it is interesting to note that some highly specialized cortical areas are known to be small, e.g., the primary hand motor area in the precentral knob^35^ or the face area in the fusiform gyrus^36^. Thus, these results suggest that the left aPFO is a highly specialized area, which was confirmed with the analysis of its FC pattern.

An important result is indeed the discovery that aPFO in the left hemisphere appears to be a functional hub connecting premotor regions involved in speech processing (i.e., pre-SMA, DMPFC, area 55b) with the ventral and the dorsal fronto-temporal language networks, involved, respectively, in the processing of semantic (mapping sound-to-meaning) and phonologic (mapping sound-to-articulation) information^37^. So far, these two latter networks were thought to find their frontal cortical origins in segregated regions. Indeed, (1) the dorsal network refers to the arcuate fasciculus (AF) connecting area 44 with the dorsal posterior temporal region (Wernicke’s area), and (2) the ventral pathway refers to the temporo-frontal extreme capsule fasciculus (TFexcF) connecting area 45 with the anterior parts of the superior and middle temporal gyri (i.e., the parts anterior to the sulcus acousticus) via the extreme capsule^38–43^. An important finding here is the discovery of a region in the left hemisphere that allows the integration of both networks, thus rendering possible the integration of phonological and semantic information. Furthermore, our results show that pPFO belongs to the cognitive control network. This is consistent with neuroimaging studies that have assigned a role to pPFO, bilaterally, in performance monitoring, especially in the analysis of external and internal feedback (positive or negative) to guide adaptive choices in non-routine environments^14,44–47^. Whether this performance monitoring processing is performed selectively by pPFO or aPFO or both remains to be determined, but our results suggest that pPFO is the key region involved. Importantly, results strongly suggest that pPFO in the left hemisphere might be an interface between the networks originating from aPFO and PCO. Note that it is unlikely that the increased FC between pPFO and aPFO on the one hand, and with PCO on the other, is due to the anatomical proximity between these regions. Indeed, if closer regions were systematically functionally connected, results should have revealed increased FC between other adjacent areas, such as aPFO and the anterior insula, or pPFO and Area 44, which is not the case. Rather, our data suggest that pPFO is a region that coordinates 2 separate networks, involving specifically and respectively aPFO and PCO. These data are in line with recent studies collectively showing that sharp transitions between functional networks relying on adjacent anatomical regions are observed in association cortices such as frontal, parietal, and temporal areas^48–50^. Thus, the left pPFO may exert a top-down control on the integration of phonological and semantic information performed by aPFO, which constitutes a crucial aspect of overt speech preparation^51^ as well as inner speaking and thinking^52^. In line with these results, a recent study has identified PFO in the left hemisphere as the region with the highest decoding accuracy for the onset of auditory verbal hallucinations (AVH) in schizophrenia patients^53^, AVH being derived from a misattribution of inner speech^54,55^.

By contrast, results show that both aPFO and pPFO display a different FC pattern in the right hemisphere. First, aPFO in the right hemisphere lacks FC with the temporal cortex, both with its ventral and dorsal components, and with the premotor DMPFC language area. This is in line with the known strong lateralization of language processing in the left hemisphere in the human brain, as proposed by Paul Broca in the 19th century (for review see 65). Rather, aPFO displays increased FC with the adjacent premotor cortex, ventrolateral prefrontal cortex, and anterior insula. Second, in addition to its FC with the cognitive control network, pPFO in the right hemisphere displays additional FC increased in the PCC. What thus can be the function of aPFO and pPFO in the right hemisphere? In addition to its role in performance monitoring processing, the right PFO (including both aPFO and pPFO), has been suggested by neuroimaging and intracranial electroencephalography studies to be causally responsible for a more general process: the control of the switch between the default mode network and the executive control network^56–58^, possibly facilitated by the recruitment of the salience network that includes PFO^59^ and the adjacent anterior insula^60,61^ as critical nodes. Our results appear to support this hypothesis as the right pPFO, but not the right aPFO, displays increased FC with PCC, a node of the default brain network (for review, see 70).

Third, the connectivity fingerprint analysis strongly suggests that aPFO is anatomically and functionally human-specific. This is in line with the known anatomical organization of this region in the primate order^8^. Indeed, the assessment of the sulcal pattern of this region emphasized the absence of the PFO in macaques as the rostral limit of the insula remains posterior to the prefrontal cortex in macaques and barely reaches it in chimpanzee^8^. It is interesting to emphasize here the similarities and differences of the organization of the temporo-frontal extreme capsule fasciculus (TFExcF) and the AF in the macaque and human brains. Whereas the organization of TFExcF appears to be similar in human and macaque brains, the AF exhibits differences across primate species^41,62^. First, whereas in humans the AF runs from the ventrolateral prefrontal cortex to the temporo-parietal junction and turns around the posterior end of the lateral fissure to reach the middle temporal cortex, it appears not to reach this region in other non-human primates^62–64^ or weakly only in chimpanzees^65^. Second, the AF displays a strong leftward asymmetry in the human brain, but this is not the case in macaques^66^. By contrast, based on functional connectivity, we do observe, surprisingly, a potential functional macaque homolog of the human pPFO, outside of the prefrontal cortex. Specifically, this region encompasses the dorsal part of GrFO, and the ventral arcuate sulcal part of F5 (i.e., F5a)^31^ (Fig. 6D). This is interesting because F5 is a premotor area controlling hand and face/mouth movements and GrFO is anatomically connected to F5a, area 44, premotor opercular areas DO and PrCO, insula, and prefrontal areas and appears to integrate limbic inputs to control hand and face/mouth actions^31,67–69^. One could thus hypothesize that this region may exert a top-down control on the precursors of communication paths, i.e., on the gestural-mouth coordination system^70^. An alternative hypothesis would be that pPFO in humans and GrFO/F5a in macaques are, in fact, involved in different types of controls, i.e., the control of speech in humans and the control of hand/orofacial responses in macaques. In that case, it would suggest that pPFO is also human-specific. Future studies may thus identify in humans whether the homolog of F5a/GrFO presents a whole-brain FC pattern similar to pPFO and whether this region is involved in the control of hand/orofacial actions. Finally, it has been suggested that the human homolog of the frontal opercular area in macaque, located at the level of the premotor/motor cortex (i.e., PrCO^71^), is PCO^31^. Altogether, our results and the literature suggest that, although there is a precursor form of dorsal and ventral “language” fronto-temporal paths in the macaque brain, they remain -at least in part- incomplete, and there is no region integrating both. Note that it is unlikely that our inability to identify the macaque homolog of the human aPFO stemmed from methodological limitations such as MRI spatial resolution or anesthesia, as these factors would presumably have similarly affected the identification of pPFO. Nonetheless, future investigations using higher-resolution imaging in awake macaques will be necessary to 1) confirm the presence of the functional homolog of the human pPFO and the absence of aPFO and 2) identify the precise function of this homolog in macaques. Future studies assessing the putative function of pPFO in the macaque brain may benefit from the present study, which identified its anatomical target in the caudal bank of the ventralmost part of the inferior arcuate sulcus, a sulcus present in all hemispheres in the macaque brain^7^. In addition, an interesting point would be to identify the relations between pPFO and the temporal voice areas, which appear to have a critical role in the evolution of communication paths since their neurons in the macaque brain have been shown to discriminate human voice from macaque vocalizations^72^.

Finally, our previous study has shown that PFO starts to be anatomically present in a very premature form in chimpanzees as the insula is observed at the antero-posterior level of the inferior precentral sulcus (and thus at the limit between the prefrontal and the premotor cortex^7^) but do not reach the fronto-orbitalis sulcus (i.e., the homolog of the human AALF), thus preventing the formation of a complete opercularization between CIRC and AALF, the emergence of HALF and a full Broca’s complex^8^. We have also shown that chimpanzees displaying in the left hemisphere a bifurcated fronto-orbitalis sulcus that provides a configuration closer to the one observed in humans (i.e., closer to an opercularization) have increased orofacial motor control as measured by their ability to produce attention-getting sounds^8^. Importantly, the fronto-orbitalis sulcus remaining in this form even in Australopithecus^32^, it is possible that pPFO is present in more developed forms in some species in the great apes lineage or in the early phase of the human lineage. It is, however, unlikely to observe aPFO in these species, as its presence requires the presence of HALF and thus a pars triangularis that appears to emerge only in *Homo Naledi*^32^.

To conclude, this study demonstrates that the prefrontal operculum in the left hemisphere in the human brain integrates information from the cognitive control network, the dorsal, and the ventral fronto-temporal language networks. It also identifies that the anterior part of the prefrontal extent of the prefrontal operculum does not find any homolog in the macaque brain. Taken together with our previous findings^8^, this study supports the notion that the emergence of PFO may have endowed hominids with a key evolutionary advantage for the development of modern speech abilities. This is in line with neuroarcheological studies suggesting that PFO appeared exclusively in the *Homo* genus alongside advanced auditory and communication abilities. Future research should precisely characterize the contribution of the human PFO to cognitive control across speech domains, employing task-based fMRI to map correlational relationships and focused transcranial ultrasound to probe causal influence, while adopting single-subject analyses to dissociate PFO function from adjacent structures, notably the anterior insula medially and lateral areas 44/45 laterally.

## Methods

### Human experiments

#### Participants

Thirty-one healthy human participants were included in the rsfMRI experiment (12 males, 19 females; mean age of all participants 26.2 ± 4.6 years). The study was carried out in accordance with the recommendations of the Code de la Santé Publique and was approved by the “Agence Nationale de Sécurité des médicaments et des produits de santé (ANSM)” and the “Comité de Protection des Personnes (CPP) Sud-Est III” (N° EudraCT: 2015-A00897-42 and 2018-A00405-50). It also received a Clinical Trial Number (NCT03119909 and NCT03483233, see https://clinicaltrials.gov). All participants gave written informed consent.

#### Imaging acquisition

Scanning was performed on a 3T Siemens Magnetom Prisma MRI Scanner (Siemens Healthcare, Erlangen, Germany), with the Siemens 64-channels Head-Neck coil. During rs-fMRI acquisition, participants were instructed to maintain ocular fixation on a white cross presented on the center of the black screen during 10 min. We used a T2* weighted multiband and multi-echo (ME) sequence with the following parameters: TR = 1500 ms, TE1 = 16.4 ms, TE2 = 37.59 ms, TE3 = 58.78 ms, FOV = 704 × 672 mm, voxel size = 2.5 mm isotropic, and 51 slices, MB factor = 3 and GRAPPA factor = 2. We obtained a total of 400 volumes per participant. Functional imaging acquisition was preceded by a T1-weighted high-resolution anatomical scan with a MPRAGE sequence (TR = 3000 ms, TE = 2.93 ms, Flip angle 8°, FOV = 280 × 320 mm, voxel size = 0.8 mm isotropic, GRAPPA factor = 2).

### Imaging data analysis

#### Preprocessing

Preprocessing steps were realized using AFNI software (Analysis of Functional NeuroImages, version 20.2.00^73^*)*, FSL software (FMRIB Software Library, 6.0.3^74^), Python library TEDANA (TE-Dependent Analysis, version 0.0.9a1^75^), and MATLAB toolbox SPM12 *(*https://www.fil.ion.ucl.ac.uk/spm/doc/intro/). First, for each run, the three time series of the multi-echo acquisition were processed with afni_proc to correct for slice timing and rigid motion. The spatial realignment parameters were estimated on the shortest TE series and applied to all series. The TEDANA script was then used to perform a TE-dependent ICA-based denoising followed by an optimal combination of the TEs series with T2* weighted averaging. Note that the brain mask required by the TEDANA pipeline was separately computed using BET^76^ applied on the temporal mean volume of the shortest TE series. Finally, fMRI images and anatomical images were spatially linearly registered into standard MNI space. All subsequent rs-fMRI processing steps were realized with AFNI software. A temporal filtering was applied to extract the spontaneous, slowly fluctuating brain activity (0.01–0.1 Hz). Linear regression was used to remove nuisance variables (the six parameter estimates for head motion, the cerebrospinal fluid, and white matter signals from the segmentation). Finally, a spatial smoothing with a 4-mm full-width half maximum (FWHM) Gaussian kernel was then applied to the output of the regression. Note that this FWHM was chosen to strictly assess the connectivity of the frontal operculum, while excluding the adjacent insula.

#### Data-driven parcellation

The first step was to identify and manually draw a mask of the left and right frontal operculum for each individual subject based on the anatomical MRI scan. The visualization and drawing processes were executed using AFNI software. In each subject, the frontal operculum mask drawing was based on the following anatomical features (Fig. 1A), (1) the most anterior part corresponds to the junction of AALF/HALF/CIRC, (2) the most posterior part corresponds to the level of the central sulcus, and (3) laterally, the lateral cortical surface was excluded, (4) medially, the drawing stopped at the fold of the insula. Each resulting mask was resampled from anatomical resolution (voxel size = 0.8 mm isotropic) to functional resolution (voxel size = 2.5 mm isotropic). The time course of each voxel of the resulting mask (n voxels) and voxels of the whole brain cortical and subcortical gray matter (excluding the mask; m voxels) is extracted. We then calculated the Pearson’s correlation coefficients between each time series voxels in the mask and each voxel in the rest of the brain. We converted these values into Fisher’s z-scores, which created an n × m connectivity profile matrix. From this matrix, we computed the cross-correlation between the n frontal operculum voxels to generate a distance matrix. We apply spectral decomposition to the resulting distance matrix, with the number of neighbors determined as the square root of the number of voxels in the frontal operculum *√n*.

The second step determined the optimal number of clusters for each subject’s frontal operculum mask. We systematically tested clustering solutions from 2 to 5 clusters and calculated the Silhouette score (i.e., the ratio of the difference between the mean intra-cluster and nearest-cluster distances to their maximum) for each configuration. To identify the optimal cluster number that would be consistent across subjects, we computed the coefficient of variation (standard deviation divided by the mean) for each clustering solution across all subjects. The cluster number with the lowest coefficient of variation was selected, as this represented the best balance between high average Silhouette scores and homogeneous performance across the group.

All the clustering procedure was built with Python 3.8.10 and the following libraries: scikit-learn^77^ for all the clustering part, pandas^78^ for the data management, numpy^79^ for calculation and matrix building and operation, and nibabel^80^ for NiFti file management. The scripts are available on github at https://github.com/CharlesVerstraete/RSfMRI-connectivity-based-parcelisation.

#### Ipsilateral cortico-cortical FC pattern of each subregion

Once the distinct subregions of the frontal operculum were extracted in each subject and in each hemisphere, the set of ipsilateral whole-brain cerebral cortical regions displaying increased FC with the frontal operculum was identified based on the Glasser’s atlas, which is parcellated in 180 cortical ROIs^27^. Specifically, for each subject, we (1) extracted the average time course of the BOLD signal in each frontal opercular subregion and in each ROI, (2) computed the Z-score between these time courses, and (3) averaged these Z-scores across participants separately for each hemisphere. ROIs displaying a mean Z-score>0.3 (i.e., corresponding to *p* < 0.01) with at least one of the frontal operculum subregions were categorized as displaying a significant FC with the frontal operculum and isolated for further identification of which network they belong to. Discrete ROIs belonging to the same region (based on the literature) were pooled together as described in Table 1. Note that we assessed here only the cortico-cortical FC pattern of each frontal operculum subdivision, especially because the Glasser’s atlas provides a fine-grained parcellation of the cerebral cortex but not of subcortical structures.

#### Statistics and reproducibility

To identify whether the volume of the frontal operculum was similar in the left versus right hemisphere, we applied a Generalized Linear Mixed Model (GLMM). To account for both inter-individual variability and participant-specific sensitivity to frontal operculum volume, GLMM was built in the form *lmer(volume ~ hemisphere* + *(1* | *ID)* + *(0 + volume* | *ID))* (lmer package on R), allowing fixed effects of hemisphere and a random intercept per participant (ID, *N* = 31), as well as a random slope of frontal operculum volume across individuals.

To identify whether the percentage of occupation of each subregion in the entire frontal operculum mask in the left versus right hemisphere, we applied a GLMM. To account for both inter-individual variability and participant-specific sensitivity to the percentage of occupation of each subregion in the entire frontal operculum mask, GLMM was built in the form lmer(%_occupation ~ subregion*hemisphere + *(1* | *ID)* + *(0 + subregion* | *ID)*.

Results showing that the set of ROIs displaying increased FC with at least one frontal operculum subregion belongs to three different networks (i.e., language, cognitive control, and sensorimotor-auditory), we applied a GLMM separately for each hemisphere to test the effects of the frontal operculum subregions and the three networks and their interaction based on the Z-scores, with subject identity as a random factor: lmer(zscore ~ subregion*network + *1* | *ID)*. To identify whether the FC varied between (1) aPFO and pPFO, (2) aPFO and PCO, and (3) pPFO and PCO, we applied a GLMM separately for each hemisphere to test the effects of the local network based on the Z-scores, with subject identity as a random factor: *lmer(zscore ~ local_network* + *1* | *ID)*.

For each GLMM, post-hoc tests consisted in pairwise post-hoc Tukey tests (lsmeans package on R), and the extent to which fixed effects explained the variance was assessed with Marginal R² (Performance package on R). All statistics were performed with R software, R Development Core Team, under RStudio.

### Monkey experiments

#### Participants

Eighteen rhesus monkeys (*macaca mulatta*) were assessed in this study: 13 females (5–19 years old) and 5 males (10–17 years old), weighing from 5 to 13 kg. All procedures followed the European Community Council Directive (directive 2010/63/UE) (Ministère de l’Agriculture et de la Forêt, Commission nationale de l’expérimentation animale) and were approved by the local ethical committee (Comité d’Ethique Lyonnais pour les Neurosciences Expérimentales, CELYNE, C2EA #42).

#### Animal preparation

Before anesthesia, monkeys were administered glycopyrrolate (Robinul: 0.06 mg/kg), an anticholinergic agent. After a 20-min interval, anesthesia was induced with an intramuscular injection of tiletamine and zolazepam (Zoletil: 7 mg/kg). Subsequently, the animals were intubated and ventilated with oxygen-enriched air and different % Isoflurane depending on the monkeys (Table 2) throughout the entire scanning session. Note that the % of isoflurane corresponds to the standard dose commonly used in the field to maintain sufficient brain activity for resting-state fMRI while ensuring stable physiological parameters of anesthesia and preventing arousal^30,81^. To minimize variability in measurements, an MRI-compatible stereotaxic frame (Kopf, CA, USA) secured the monkeys’ heads in a sphinx position facing the back of the scanner. Breathing volume and frequency were adjusted based on the animals’ weights. Physiological parameters, including heart rate and ventilation parameters (SpO2 and CO2), were continuously monitored during the scan. Body temperature was maintained using warm air circulating blankets. RS-fMRI data acquisitions were performed approximately 2 h after anesthesia induction and at least 1 h after the initial inhalation of isoflurane.

#### rs-fRMI data acquisition

rs-fMRI data were acquired in anesthetized monkeys from a 3T Siemens Magnetom Prisma MRI scanner (Siemens Healthcare, Erlangen, Germany). Three Siemens loop coils were used: two L11 ring coils on each side of the monkey’s head and one L7 above the monkey’s head. A high-resolution T1-weighted anatomical scan (MPRAGE, 0.5 mm^3 isotropic voxels, 144 slices, TR = 3 000 ms, TE = 3.62 ms) was acquired for each of the 18 monkeys. rs-fMRI images covering the entire brain were obtained using a T2* weighted gradient echo planar imaging (EPI) sequence. The acquisition parameters varied slightly for different groups of monkeys (Table 3). For each of the macaques, five or six runs of 400 volumes each were collected. Anatomical and functional data of each animal were acquired during the same session.

**Table 3:** fMRI acquisition and anesthesia parameters for each monkey

Monkey	% isoflurane	*n* slices	TR (s)	Spatial resolution	Nb RUN
P	1%	28	1.8	1.7 mm^3	5
K	1%	31	2	1.8 mm^3	6
H	0.8%	31	1.9	1.7 mm^3	5
O	1%	31	2	1.8 mm^3	6
Po	1%	31	2	1.8 mm^3	6
Y	1.2%	31	2	1.8 mm^3	6
S	1%	25	1.7	1.7 mm^3	5
V	0.8%	28	1.8	1.7 mm^3	5
D	0.8%	28	1.8	1.7 mm^3	5
A	1%	31	2.108	1.8 mm^3	6
Ce	1.5%	31	2.108	1.8 mm^3	6
Ci	1%	31	2.108	1.8 mm^3	6
E	1%	31	2.108	1.8 mm^3	6
Ge	1%	31	2.108	1.8 mm^3	6
Gu	1%	31	2.108	1.8 mm^3	6
L	0.8%	28	1.8	1.7 mm^3	6
N	0.8%	28	1.8	1.7 mm^3	5
Pi	0.8%	28	1.8	1.7 mm^3	5

#### rs-fMRI data analysis

rs-fMRI scans were preprocessed using SPM 12. The initial 5 volumes of each run were excluded to account for T1 equilibrium effects. Slice timing correction was performed with the time center of the volume as a reference, followed by rigid body realignment for head motion correction. Skull-stripping was executed using the bet tool from FSL software^74^. AFNI software^73^ was then employed for brain segmentation on the previously skull-stripped brains. Anatomical and functional data were then registered to the CHARM common atlas space^82^. Temporal filtering extracted spontaneous slow fluctuating brain activity within the 0.01–0.1 Hz range. Linear regression removed nuisance variables, including cerebrospinal fluid and white matter signals from segmentation, as well as volumes with detected artifacts using the ART toolbox (https://www.nitrc.org/projects/artifact_detect/). No spatial smoothing was applied to the regression output for the connectivity fingerprint analysis.

#### Connectivity fingerprint analysis

ROIs in the macaque brain anatomically homologous to those observed in the human spiders (Fig. 3), corresponding to the ipsilateral FC pattern of aPFO and pPFO, were identified from level 6 of the CHARM atlas. Table 2 summarizes the corresponding regions and discrete ROIs from the CHARM atlas involved.

#### Connectivity fingerprint matching

Connectivity fingerprint matching^23^ provides a non-invasive approach to match a particular region across different brains based on its unique connectivity pattern. This method is based on the idea that any brain region can be uniquely defined by a set of important connections it has with other regions, which is essential for accomplishing its function^12^. In the present study, we adopted this method to identify the potential homologs of the aPFO, pPFO, and PCO in the macaque brain based on the connectivity fingerprints of these regions in the human brain.

The mean connectivity fingerprints of the human aPFO and pPFO were first computed by averaging the individual fingerprints of the three regions across participants. For each mean connectivity fingerprint, the connectivity strengths (seed-target correlations) are scaled to the maximum and minimum values in the fingerprint. Next, 18 ROIs in the macaque brain homologous to the ROIs observed in the human aPFO, and pPFO connectivity fingerprints (Fig. 3) were identified from the level 6 of the CHARM atlas (see Table 2). Note that (1) human area 55b was not used in macaques as an ROI, as it does not find a correspondence in the CHARM atlas, (2) human STSa and STSp are 2 separate ROIs, but in macaques, the STS CHARM ROI encompasses both STSa and STSp. This latter point is not an issue, as FC between aPFO and STSa is similar to the FC with STSp. Table 2 summarizes the corresponding regions and discrete ROIs from the CHARM atlas involved. In each monkey, each hemisphere, and each run, we first computed the correlation between the mean signal in each of the 18 ROIs with every other voxel in the cerebral cortex (excluding subcortical structures and cerebellar cortex) using tool^83^. For each monkey, we then averaged across runs the resulting 18 whole-brain correlation maps that depicted the connectivity of each ROI with each other voxel in the brain. The 18 correlation maps obtained in the 18 monkeys were then concatenated in the “time” dimension into a single 4D matrix. In this 4D matrix, each voxel thus contained 18 connectivity values in the “time” dimension that indicated the connectivity between that voxel and each of the 18 ROIs for each monkey. Finally, permutation testing, via *FSL randomize*^84^, was performed to determine which voxels in each monkey’s concatenated correlation maps show a statistically significant GLM fit in their pattern of connectivity with the mean connectivity fingerprints of the human aPFO and pPFO. For the permutation test, the sampling distribution was derived by shuffling 5000 times the order of the 18 correlation maps and by re-computing the statistical test. We assessed the significance of the results at *p* < 0.05 Family-Wise Error (FWE) corrected.

### Reporting summary

Further information on research design is available in the Nature Portfolio Reporting Summary linked to this article.

## Supplementary information

Supplementary Information

Description of Additional Supplementary Files

Supplemental Data 1

Supplemental Data 2

Supplemental Data 3

Supplemental Data 4

Supplemental Data 5

Supplemental Data 6

Reporting Summary

## Data Availability

The source data underlying all figures are provided as a Source Supplemental Data. Supplementary Data 1 provides the probabilistic maps of the frontal operculum mask in the left and right hemispheres as presented in Fig. 1 (zip file). Supplementary Data 2 provides the probabilistic maps of the 3 subdivisions composing the frontal operculum mask (i.e., aPFO, pPFO, PCO) in the left and right hemisphere as presented in Fig. 2A, B, C (zip file). Supplementary Data 3 provides the source data behind the boxplots in Fig. 2D (xlsx file). Supplementary Data 4 provides the average functional connectivity patterns across 31 subjects of aPFO, pPFO, and PCO in the left and right hemispheres as presented in Fig. 3A et 4 A (zip file). Supplementary Data 5 provides the source data behind the spider plots presented in Figs. 3C and 4C (xlsx file). Supplementary Data 6 provides the source data behind the boxplots presented in Fig. 5 (xlsx file). Macaque anatomical and resting-state fMRI scans are available from the PRIMatE Data Exchange (PRIME-DE) database [https://fcon_1000.projects.nitrc.org/indi/indiPRIME.html]. A reporting summary for this Article is available as a Supplementary Information file.
