# Supplementary material for: The prefrontal operculum, a human-specific hub for the cognitive control of speech
Source: Commun Biol. 2025 Dec 1;8:1731. doi: 10.1038/s42003-025-09110-8 (PMC12669689; doi:10.1038/s42003-025-09110-8)
Supplement: Supplementary file 1 — Supplementary Information [file 42003_2025_9110_MOESM1_ESM.pdf]

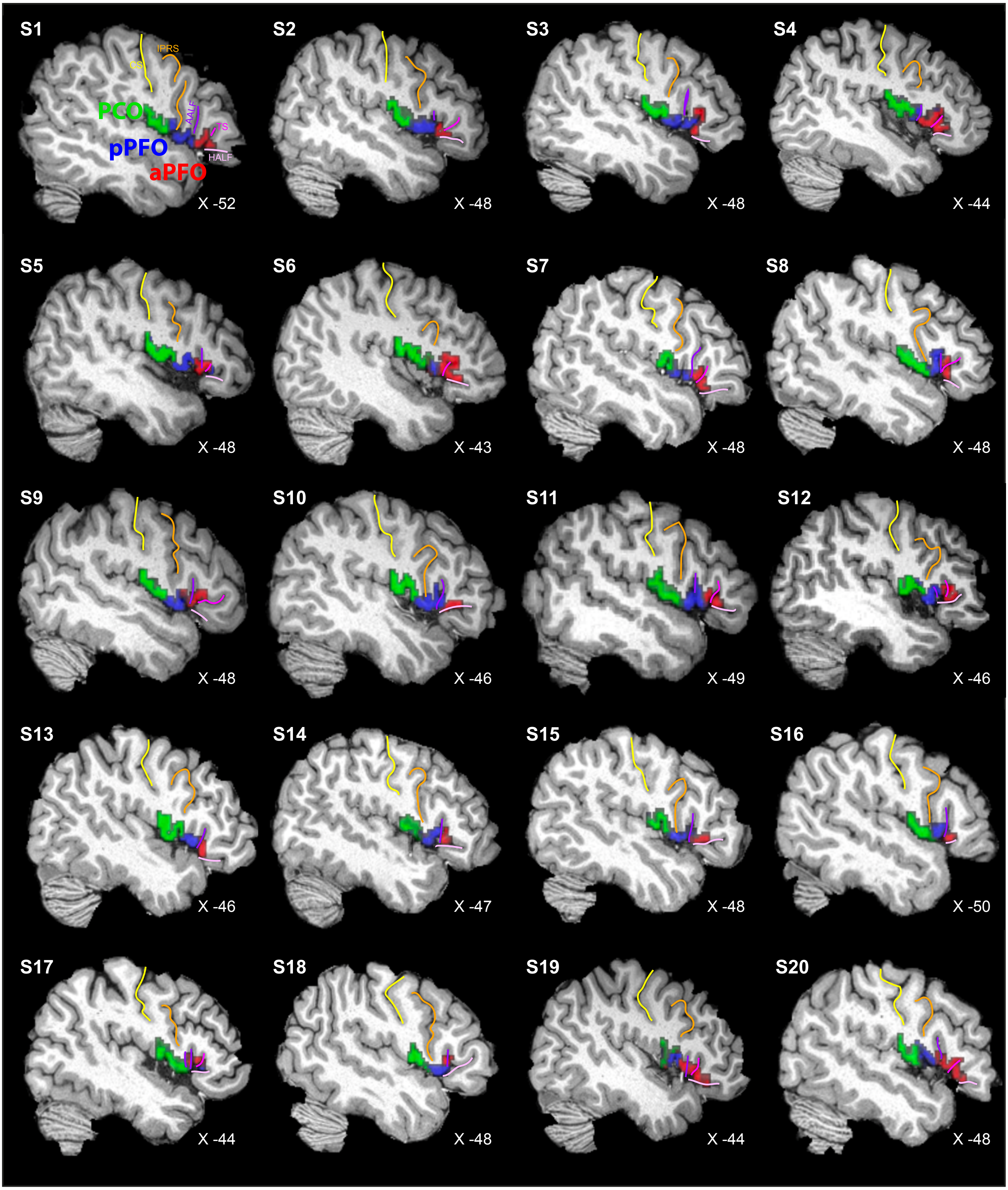
S1
IPRS
CS
PCO
AALF
TS
pPFO
HALF
aPFO
X -52
S2
X -48
S3
X -48
S4
X -44
S5
X -48
S6
X -43
S7
X -48
S8
X -48
S9
X -48
S10
X -46
S11
X -49
S12
X -46
S13
X -46
S14
X -47
S15
X -48
S16
X -50
S17
X -44
S18
X -48
S19
X -44
S20
X -48

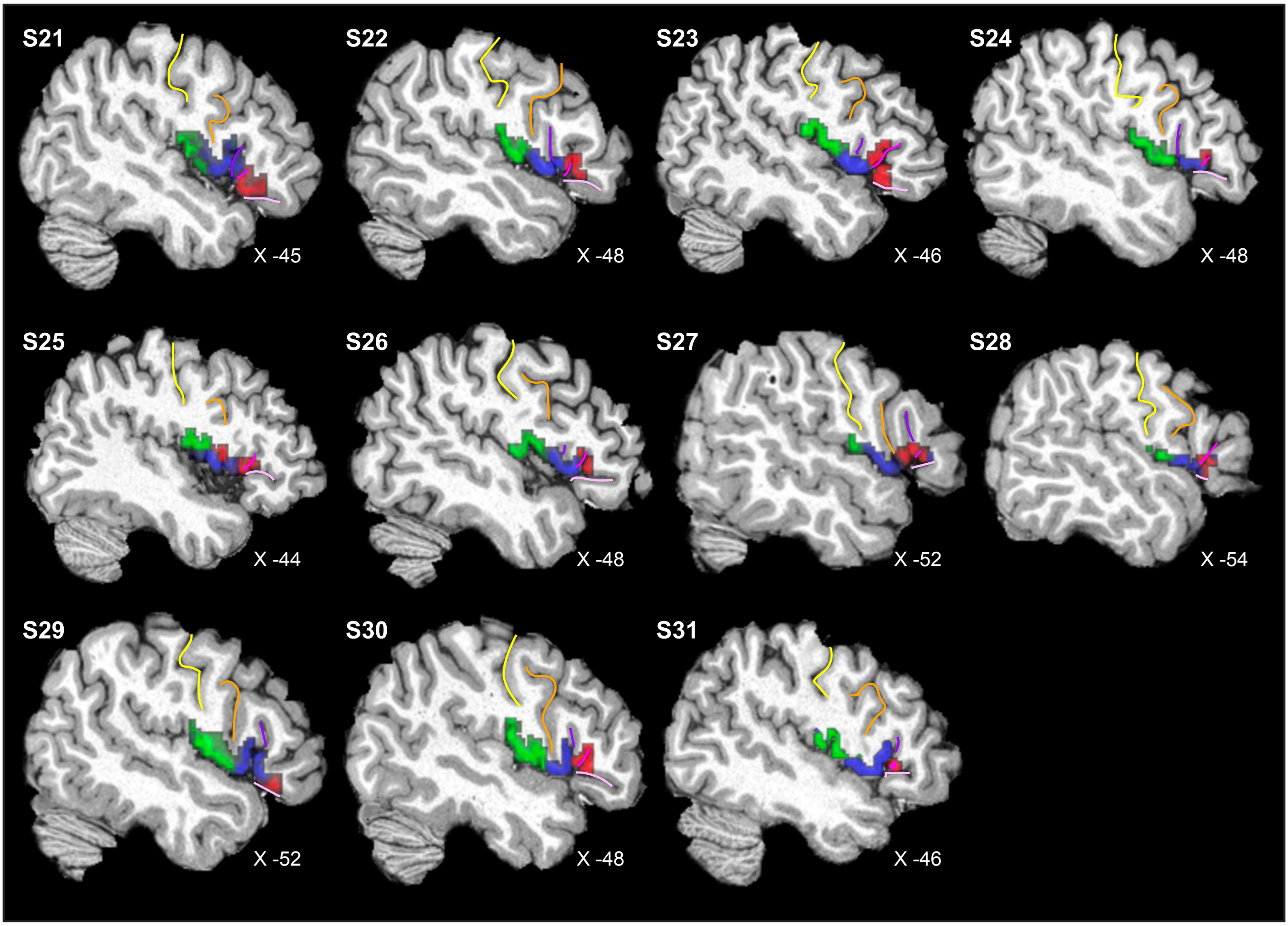

Fig S1. Location of aPFO (red, anterior part), pPFO (blue, mid part), and PCO (green, posterior part) in all subjects in the left hemisphere. They are presented on the corresponding anatomical scan of each subject registered in the MNI space. X values represent the medio-lateral levels of the presented slices in the standard MNI space.

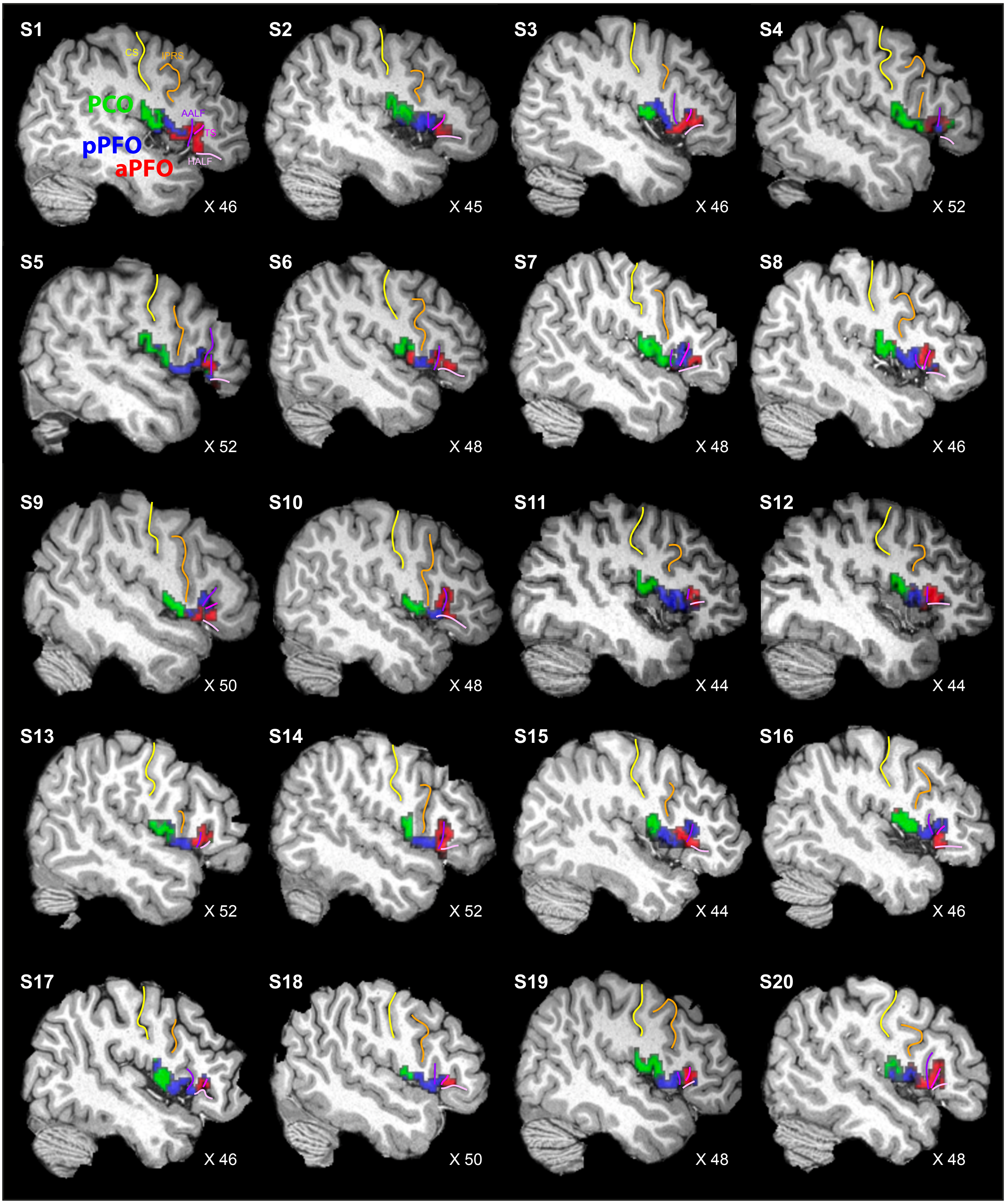

S1
CS
IPRS
PCO
AALF
TS
pPFO
aPFO
HALF
X 46
S2
X 45
S3
X 46
S4
X 52
S5
X 52
S6
X 48
S7
X 48
S8
X 46
S9
X 50
S10
X 48
S11
X 44
S12
X 44
S13
X 52
S14
X 52
S15
X 44
S16
X 46
S17
X 46
S18
X 50
S19
X 48
S20
X 48

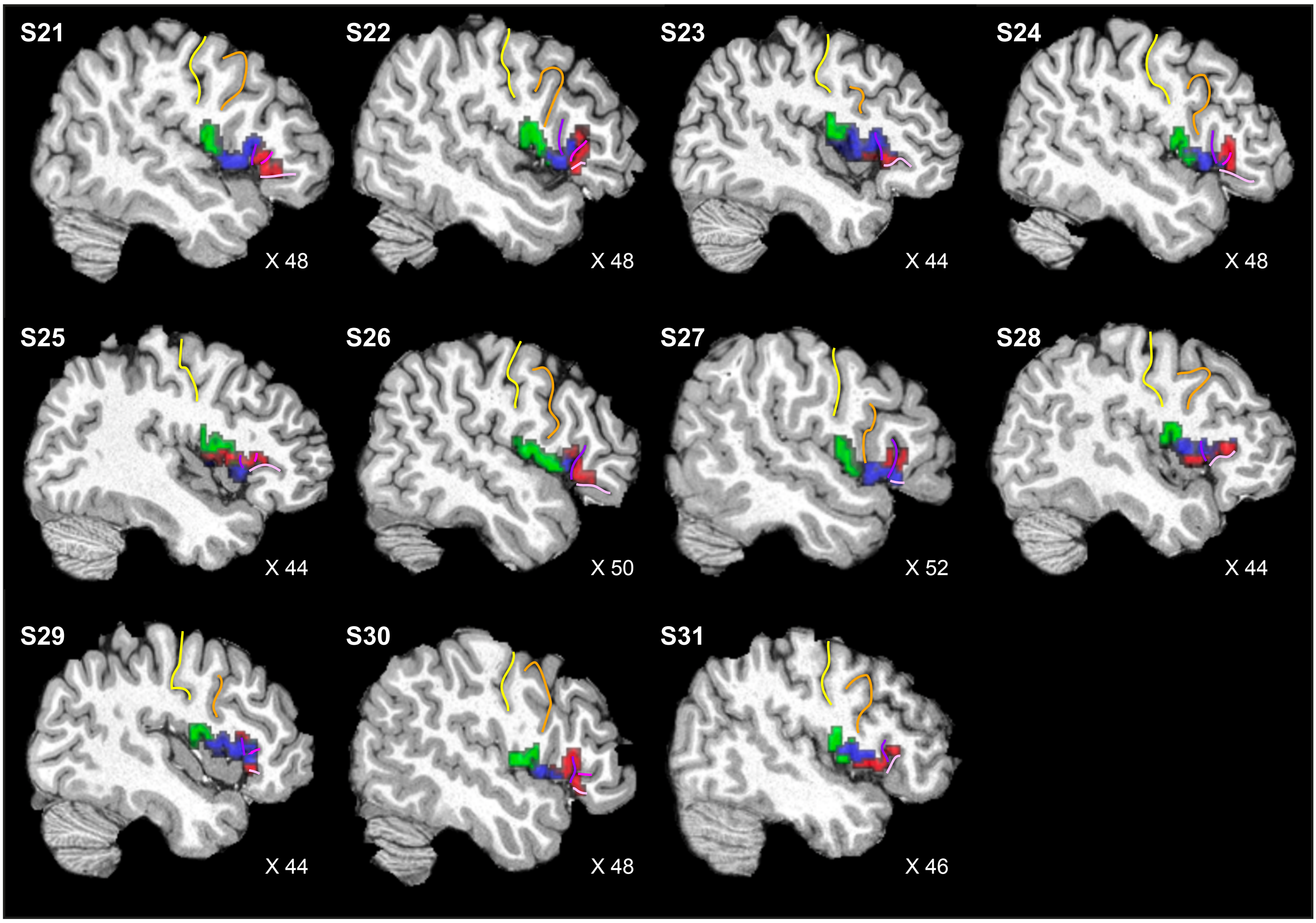

Fig S2. Location of aPFO (red, anterior part), pPFO (blue, mid part), and PCO (green, posterior part) in all subjects in the right hemisphere. They are presented on the corresponding anatomical scan of each subject registered in the MNI space. X values represent the medio-lateral levels of the presented slices in the standard MNI space.

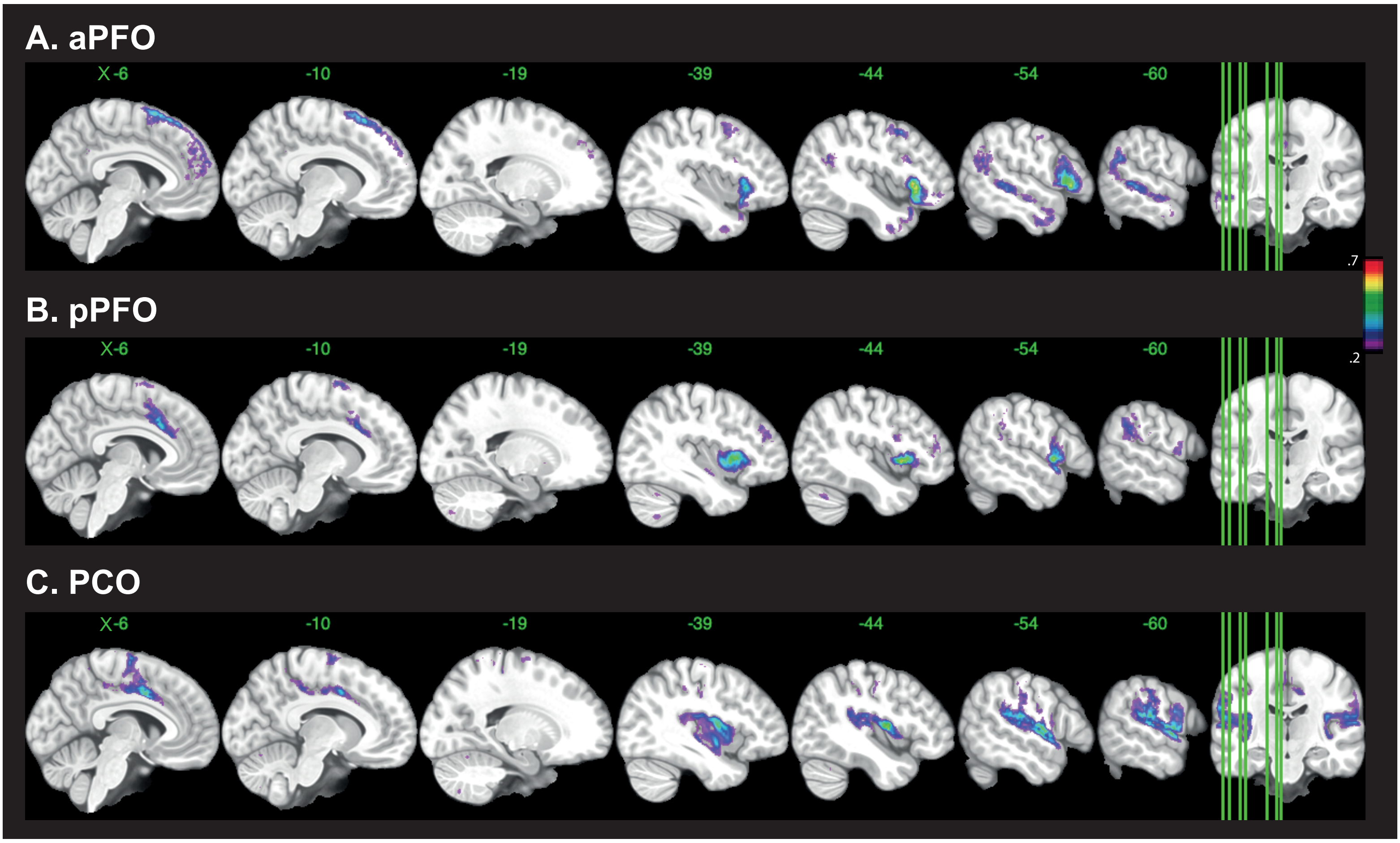

Figure S3. Left ipsilateral FC patterns of aPFO (A), pPFO (B), and PCO (C) across 31 subjects. FC patterns are displayed on the non-linear anatomical MNI template and thresholded at Z=0.22 (p<0.01). X values represent the medio-lateral level of the sagittal slice. The Z -values range is represented by the color bar.

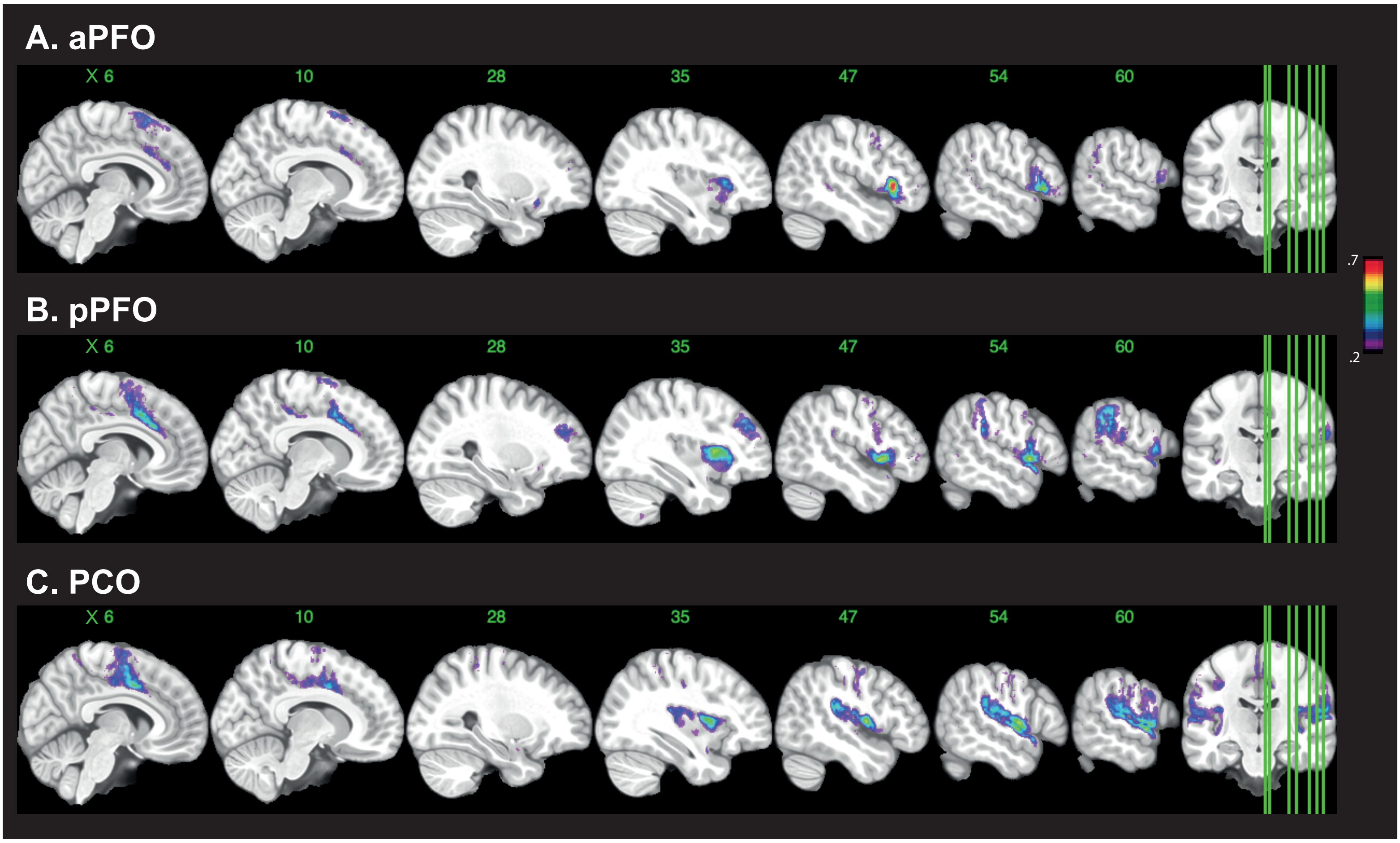

Figure S4. Right ipsilateral FC patterns of aPFO (A), pPFO (B), and PCO (C) across 31 subjects. FC patterns are displayed on the non-linear anatomical MNI template and thresholded at Z=0.22 (p<0.01). X values represent the medio-lateral level of the sagittal slice. The Z -values range is represented by the color bar.
