# Supplementary material for: The prefrontal operculum, a human-specific hub for the cognitive control of speech
Source: Commun Biol. 2025 Dec 1;8:1731. doi: 10.1038/s42003-025-09110-8 (PMC12669689; doi:10.1038/s42003-025-09110-8)
Supplement: Supplementary file 2 — Description of Additional Supplementary Files [file 42003_2025_9110_MOESM2_ESM.docx]

**Description of Additional Supplementary files**

File name: Supplementary Data 1

Description: Probabilistic map of frontal operculum mask in the left and right hemisphere as presented in Fig 1 (zip file).

File name: Supplementary Data 2

Description: Probabilistic map of the 3 subdivisions composing the frontal operculum mask (i.e. aPFO, pPFO, PCO) in the left and right hemisphere as presented in Fig 2A, B, C (zip file).

File name: Supplementary Data 3

Description: The source data behind the graphs in Fig 2D (xlsx file).

File name: Supplementary Data 4

Description: Average FC patterns across 31 subjects of aPFO, pPFO, PCO in the left and right hemispheres as presented in Fig 3A et 4A (zip file).


File name: Supplementary Data 5

Description: The source data behind the spiders plots presented in Fig 3C and 4C (xlsx file)

File name: Supplementary Data 6

Description: Description: The source data behind the graphs in Fig 5 (xlsx file).
