# Supplementary material for: The prefrontal operculum, a human-specific hub for the cognitive control of speech
Source: Commun Biol. 2025 Dec 1;8:1731. doi: 10.1038/s42003-025-09110-8 (PMC12669689; doi:10.1038/s42003-025-09110-8)
Supplement: Supplementary file 9 — Reporting Summary [file 42003_2025_9110_MOESM9_ESM.pdf]

nature portfolio

Corresponding author(s): Céline Amiez; Charles Verstraete

Last updated by author(s): 2025/06/25

Reporting Summary

Nature Portfolio wishes to improve the reproducibility of the work that we publish. This form provides structure for consistency and transparency in reporting. For further information on Nature Portfolio policies, see our Editorial Policies and the Editorial Policy Checklist.

Statistics

For all statistical analyses, confirm that the following items are present in the figure legend, table legend, main text, or Methods section.

n/a	Confirmed	
☐	☒	The exact sample size (*n*) for each experimental group/condition, given as a discrete number and unit of measurement
☐	☒	A statement on whether measurements were taken from distinct samples or whether the same sample was measured repeatedly
☐	☒	The statistical test(s) used AND whether they are one- or two-sided<br>*Only common tests should be described solely by name; describe more complex techniques in the Methods section.*
☒	☐	A description of all covariates tested
☐	☒	A description of any assumptions or corrections, such as tests of normality and adjustment for multiple comparisons
☐	☒	A full description of the statistical parameters including central tendency (e.g. means) or other basic estimates (e.g. regression coefficient) AND variation (e.g. standard deviation) or associated estimates of uncertainty (e.g. confidence intervals)
☐	☒	For null hypothesis testing, the test statistic (e.g. F, t, r) with confidence intervals, effect sizes, degrees of freedom and P value noted<br>*Give P values as exact values whenever suitable.*
☒	☐	For Bayesian analysis, information on the choice of priors and Markov chain Monte Carlo settings
☒	☐	For hierarchical and complex designs, identification of the appropriate level for tests and full reporting of outcomes
☒	☐	Estimates of effect sizes (e.g. Cohen's *d*, Pearson's *r*), indicating how they were calculated

Our web collection on statistics for biologists contains articles on many of the points above.

Software and code

Policy information about availability of computer code

Data collection	NA
Data analysis	Preprocessing steps of resting state fMRI data were realized using AFNI software (Analysis of Functional NeuroImages), FSL software (FMRIB Software Library), Python library TEDANA (TE-Dependent Analysis,) and MATLAB toolbox SPM12 (https://www.fil.ion.ucl.ac.uk/spm/software/spm12/).<br>All the clustering procedure was built with Python 3.8.10 and the following libraries: scikit-learn for all the clustering part, pandas for the data management, numpy for calculation and matrix building and operation, and nibabel for NiFti file management. The scripts are available on github at https://github.com/CharlesVerstraete/RSfMRI-connectivity-based-parcalisation.<br>All statistics regarding human data were performed with R software, R Development Core Team under R-Studio.<br>The connectivity fingerprint matching analysis in macaques was performed with FSL according to the method developed by Mars et al. (2016).

For manuscripts utilizing custom algorithms or software that are central to the research but not yet described in published literature, software must be made available to editors and reviewers. We strongly encourage code deposition in a community repository (e.g. GitHub). See the Nature Portfolio guidelines for submitting code & software for further information.

Data

Policy information about availability of data

All manuscripts must include a data availability statement. This statement should provide the following information, where applicable:

- Accession codes, unique identifiers, or web links for publicly available datasets
- A description of any restrictions on data availability
- For clinical datasets or third party data, please ensure that the statement adheres to our policy

The source data underlying all Figures are provided as a Source Data file. Macaque anatomical and resting-state fMRI scans are available from the PRIMatE Data Exchange (PRIME-DE) database [http://fcon_1000.projects.nitrc.org/indi/indiPRIME.html]. A reporting summary for this Article is available as a Supplementary Information file.

Research involving human participants, their data, or biological material

Policy information about studies with human participants or human data. See also policy information about sex, gender (identity/presentation), and sexual orientation and race, ethnicity and racism.

Reporting on sex and gender	Thirty-one healthy human subjects participated in the rsfMRI experiment: 19 females, 12 males.
Reporting on race, ethnicity, or other socially relevant groupings	NA
Population characteristics	Thirty-one healthy human subjects participated in the rsfMRI experiment. Mean age of all subjects was 26.2 ± 4.6 years.
Recruitment	Healthy adult volunteers were recruited through postings in universities, laboratories and hospital departments, or on the proposal of the investigator or scientific collaborators.
Ethics oversight	The study was carried out in accordance with the recommendations of the Code de la Santé Publique and was approved by the “Agence Nationale de Sécurité des médicaments et des produits de santé (ANSM)” and the “Comité de Protection des Personnes (CPP) Sud-Est III” (N° EudraCT: 2015-A00897-42 and 2018-A00405-50). It also received a Clinical Trial Number (NCT03119909 and NCT03483233, see https://clinicaltrials.gov). All subjects gave written informed consent.

Note that full information on the approval of the study protocol must also be provided in the manuscript.

Field-specific reporting

Please select the one below that is the best fit for your research. If you are not sure, read the appropriate sections before making your selection.

☒ Life sciences ☐ Behavioural & social sciences ☐ Ecological, evolutionary & environmental sciences

For a reference copy of the document with all sections, see nature.com/documents/nr-reporting-summary-flat.pdf

Life sciences study design

All studies must disclose on these points even when the disclosure is negative.

Sample size	31 human subjects, 18 macaques.
Data exclusions	All subjects were included
Replication	NA
Randomization	Randomization is not pertinent in our study. Human brains and macaque brains are treated separately.
Blinding	Blinding across group is also not relevant, primate brains being analysed separately.

Reporting for specific materials, systems and methods

We require information from authors about some types of materials, experimental systems and methods used in many studies. Here, indicate whether each material, system or method listed is relevant to your study. If you are not sure if a list item applies to your research, read the appropriate section before selecting a response.

Materials & experimental systems

n/a	Involved in the study
☒	☐ Antibodies
☒	☐ Eukaryotic cell lines
☒	☐ Palaeontology and archaeology
☐	☒ Animals and other organisms
☒	☐ Clinical data
☒	☐ Dual use research of concern
☒	☐ Plants

Methods

n/a	Involved in the study
☒	☐ ChIP-seq
☒	☐ Flow cytometry
☒	☐ MRI-based neuroimaging

Animals and other research organisms

Policy information about studies involving animals; ARRIVE guidelines recommended for reporting animal research, and Sex and Gender in Research

Laboratory animals: Eighteen rhesus monkeys (macaca mulatta) were assessed in this study: 13 females and 5 males.

Wild animals: No

Reporting on sex: Eighteen rhesus monkeys (macaca mulatta) were assessed in this study: 13 females (5-19 years old) and 5 males (10-17 years old) weighing from 5 to 13 kg.

Field-collected samples: NA

Ethics oversight: All procedures followed the European Community Council Directive (directive 2010/63/UE) (Ministère de l'Agriculture et de la Forêt, Commission nationale de l'expérimentation animale) and were approved by the local ethical committee (Comité d'Ethique Lyonnais pour les Neurosciences Expérimentales, CELYNE, C2EA #42).

Note that full information on the approval of the study protocol must also be provided in the manuscript.

Plants

Seed stocks: *Report on the source of all seed stocks or other plant material used. If applicable, state the seed stock centre and catalogue number. If plant specimens were collected from the field, describe the collection location, date and sampling procedures.*

Novel plant genotypes: *Describe the methods by which all novel plant genotypes were produced. This includes those generated by transgenic approaches, gene editing, chemical/radiation-based mutagenesis and hybridization. For transgenic lines, describe the transformation method, the number of independent lines analyzed and the generation upon which experiments were performed. For gene-edited lines, describe the editor used, the endogenous sequence targeted for editing, the targeting guide RNA sequence (if applicable) and how the editor was applied.*

Authentication: *Describe any authentication procedures for each seed stock used or novel genotype generated. Describe any experiments used to assess the effect of a mutation and, where applicable, how potential secondary effects (e.g. second site T-DNA insertions, mosiacism, off-target gene editing) were examined.*
